# Multi-scale account of the network structure of macaque visual cortex

**DOI:** 10.1007/s00429-017-1554-4

**Published:** 2017-11-16

**Authors:** Maximilian Schmidt, Rembrandt Bakker, Claus C. Hilgetag, Markus Diesmann, Sacha J. van Albada

**Affiliations:** 1 Institute of Neuroscience and Medicine (INM-6) and Institute for Advanced Simulation (IAS-6) and JARA Institute Brain Structure-Function Relationships (JBI-1 /INM-10), Jülich Research Centre, Jülich, Germany; 2 Donders Institute for Brain, Cognition and Behavior, Radboud University Nijmegen, Nijmegen, The Netherlands; 3 Institute of Computational Neuroscience, University Medical Center Eppendorf, Hamburg, Germany; 4 Department of Health Sciences, Boston University, Boston, USA; 5 Department of Psychiatry, Psychotherapy and Psychosomatics, Medical Faculty, RWTH Aachen University, Aachen, Germany; 6 Department of Physics, Faculty 1, RWTH Aachen University, Aachen, Germany

**Keywords:** Macaque visual cortex, Cellular architecture, Cortical layers, Multi-scale connectivity, Predictive connectomics

## Abstract

Cortical network structure has been extensively characterized at the level of local circuits and in terms of long-range connectivity, but seldom in a manner that integrates both of these scales. Furthermore, while the connectivity of cortex is known to be related to its architecture, this knowledge has not been used to derive a comprehensive cortical connectivity map. In this study, we integrate data on cortical architecture and axonal tracing data into a consistent multi-scale framework of the structure of one hemisphere of macaque vision-related cortex. The connectivity model predicts the connection probability between any two neurons based on their types and locations within areas and layers. Our analysis reveals regularities of cortical structure. We confirm that cortical thickness decays with cell density. A gradual reduction in neuron density together with the relative constancy of the volume density of synapses across cortical areas yields denser connectivity in visual areas more remote from sensory inputs and of lower structural differentiation. Further, we find a systematic relation between laminar patterns on source and target sides of cortical projections, extending previous findings from combined anterograde and retrograde tracing experiments. Going beyond the classical schemes, we statistically assign synapses to target neurons based on anatomical reconstructions, which suggests that layer 4 neurons receive substantial feedback input. Our derived connectivity exhibits a community structure that corresponds more closely with known functional groupings than previous connectivity maps and identifies layer-specific directional differences in cortico-cortical pathways. The resulting network can form the basis for studies relating structure to neural dynamics in mammalian cortex at multiple scales.

**Electronic supplementary material:**

The online version of this article (10.1007/s00429-017-1554-4) contains supplementary material, which is available to authorized users.

## Introduction

Connectivity maps allow insights into the structure of the brain, for instance through graph-theoretical analyses (Jouve et al. 1998; Rubinov and Sporns 2010), and help to create hypotheses on neural processing strategies (Maunsell and Newsome 1987; Felleman and Van Essen 1991; Nassi and Callaway 2009). For instance, experimental knowledge about laminar patterns of connectivity (Felleman and Van Essen 1991; Markov et al. 2014b) in combination with experimental studies on cortical activity (van Kerkoerle et al. 2014; Bastos et al. 2015a) have inspired theories about hierarchical processing and communication between cortical areas (Bastos et al. 2012, 2015b). Furthermore, connectivity maps provide a structural basis for dynamical models of the brain. They have been derived at different levels of detail and for different species such as the mouse (Oh et al. 2014) and macaque monkey (Stephan et al. 2001; Bakker et al. 2012). Such maps inherently possess uncertainties, for example, due to gaps in the experimental data or deformations associated with the mapping to standard brains. Consequently, there is an ongoing need for improvement, gradual refinement, and theoretical integration.

The connectivity of the brain is closely linked to its cellular architecture. Systematic relations have been identified in cortex using the notion of architectural types (Barbas 1986; Barbas and Rempel-Clower 1997), which classify the distinctiveness of the laminar cortical architecture as well as the thickness of the granular layer (Dombrowski et al. 2001). A set of connectivity features, including the existence or absence of connections and laminar patterns of cortico-cortical connections, are linked to structural differences between areas (Barbas and Rempel-Clower 1997; Hilgetag and Grant 2010; Hilgetag et al. 2016; Beul et al. 2017). The concept of architectural types represents a discretization of a continuum of structural features across areas (von Economo and Van Bogaert 1927). Types relate also to neuron density, as types with low ordinal number have low overall neuron density. Statistical relationships between cortical architecture and connectivity may have a developmental origin, with areas of low type developing earlier and having a larger time window for interconnecting with other areas (Barbas and García-Cabezas 2016; Beul et al. 2017). Regardless of the underlying cause, such regularities help to fill gaps in existing connectivity maps.

Network science describes the connectivity of neuronal networks in different ways, for instance in terms of total numbers of synapses, pairwise connection probabilities, or in- and outdegrees of nodes, but also by more abstract measures of connection strength (Hagmann et al. 2007; Wedeen et al. 2008). Some of these different measures of connectivity are related through neural population sizes, for instance, average indegrees are obtained by dividing the total number of synapses by the size of the target population. Knowledge about the cellular architecture of the brain thus allows researchers to translate between different measures of connectivity. Furthermore, combining network connectivity with a quantification of the cellular architecture leads to a cellular-level network description, necessary for dynamical model simulations at this resolution.

In the present study, we investigate the network of vision-related areas of macaque cortex, a system that has garnered intense interest in experimental studies (e.g., De Valois et al. 1982; Luck et al. 1997; van Kerkoerle et al. 2014; Bastos et al. 2015a). The available experimental data on the cellular architecture and connectivity of the system are extensive, yet still incomplete. However, structural relations and distances between areas expose statistical regularities that we employ to bridge some of the missing data.

The microcircuit model of Potjans and Diesmann (2014), which constitutes a synthesis of local connectivity data from electrophysiological and anatomical studies, forms the basis for the intra-area connectivity in our network. Although the data originate mainly from studies on rat somatosensory and cat primary visual cortex, the comprehensive collation of local connectivity by this model is unparalleled for macaque cortex, let alone for the individual areas we consider. Our choice is justified by predominant similarities between the local cortical connectivity in different species and areas, as formalized by the concept of a ‘canonical microcircuit’ (Douglas et al. 1989; Douglas and Martin 2004). We nevertheless take into account variability across areas as resulting from known differences in laminar compositions and their degree of connectivity.

The connectivity between areas in our model combines information from a recent release of the CoCoMac connectivity database (Stephan et al. 2001; Bakker et al. 2012) with quantitative data on cortico-cortical connection densities (Markov et al. 2014a) and laminar patterns (Markov et al. 2014b). For long-distance connections, tracing data are more reliable than diffusion MRI (Thomas et al. 2014), which enters into most current multi-area modeling work (Deco and Jirsa 2012; Sanz Leon et al. 2013; Kunze et al. 2016). The observed exponential fall-off of connection density with spatial distance (Ercsey-Ravasz et al. 2013) helps to estimate connection densities for area pairs where quantitative data are lacking. The categorization of areas into architectural types predicts cell densities and laminar thicknesses in case of missing data. Such structural differences between areas are in turn linked to and help fill in laminar patterns of cortico-cortical projections. A unique feature of our connectivity map is that it enables layer-specific polysynaptic pathways to be characterized, as synapse locations are statistically mapped (based on morphological reconstructions; Binzegger et al. 2004) to the locations of the target cell bodies forwarding the synaptic input. In this study, we aim to derive a consistent picture of the connectivity within and between vision-related areas within one hemisphere of macaque cortex. A treatment of callosal and subcortical connections therefore lies beyond the scope of the current study, but represents an important extension for a future revision of the model.

Besides uncovering layer-specific pathways for routing cortico-cortical communication, the resulting network description reveals a modular structure that resembles a functional categorization of areas. The derivations of the connectivity and the numbers of neurons necessarily entail choices which, given the available data, yield a compromise between detail and conciseness. Due to these simplifying assumptions and presently unexplained biological variability, the entries of the resulting connectivity matrix are only estimated up to a certain precision, and therefore the individual entries should be interpreted with care. The advantage of our approach is that it makes the assumptions explicit, which enables their consequences to be studied in a systematic manner. Furthermore, the matrix as a whole already provides a multi-scale connectivity substrate for the investigation of cortical dynamics via analytical theory and numerical simulation in a way that an incomplete matrix cannot, and various validations demonstrate the plausibility of its community structure and layer-specific pathways.

The remainder of this paper is organized as follows. In the “Materials and methods” section, we provide an overview of the processing of the experimental data contributing to the model. In the “Results” section, we detail the derivation of the network description including population sizes and the multi-scale cortical connectivity. Subsequently, we analyze the resulting connectivity map with regard to community structure and emerging paths in the network. In terms of source and target layers, we find that feedforward paths follow a stereotypical pattern, also shared by lateral paths, while feedback paths feature a high degree of heterogeneity. However, in pathways passing through several areas, the intermediate laminar patterns of lateral paths more closely resemble those of feedback paths. Finally, we discuss the implications of our results and suggest future directions in the "Discussion" section. Preliminary results have been published as preprint in Schmidt et al. (2016).

## Materials and methods

**Fig. 1:**
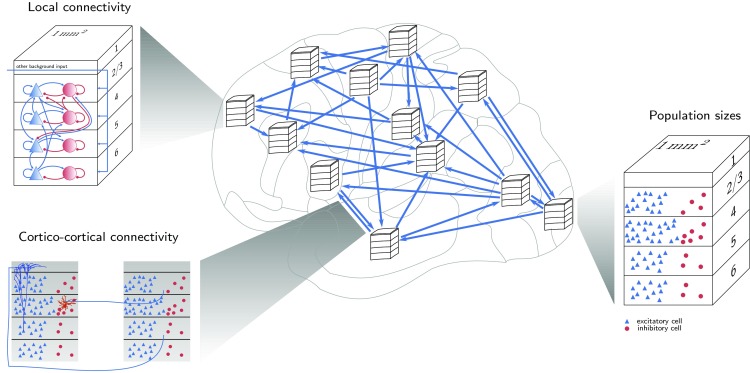
Overview of the model. Each area is modeled as the volume under $1\,\mathrm {mm}^{2}$ of cortical surface with area- and layer-specific population sizes. The local connectivity inside each area is based on the microcircuit model of Potjans and Diesmann (2014). Cortico-cortical connectivity is area- and layer-specific. It is derived from tracing data stored in the CoCoMac database (Stephan et al. 2001; Bakker et al. 2012), quantitative retrograde tracing data from Markov et al. (2014a, b) and reconstructed morphologies from Binzegger et al. (2004). Microcircuit diagrams adapted from Potjans and Diesmann (2014) (with permission). Large-scale network diagram adapted from Kunkel et al. (2009). The dendritic morphologies in the cortico-cortical connectivity illustration are extracted from Stepanyants et al. (2008) (inhibitory L4 cell) and Mainen and Sejnowski (1996) (L5 pyramidal cell), respectively (source: http://NeuroMorpho.org; Ascoli et al. 2007)

We consider a network comprising 32 areas of macaque cortex involved in visual processing in the parcellation of Felleman and Van Essen (1991), henceforth referred to as FV91 (Supplementary Table S1). In each area, we consider a microcircuit under $1\,\mathrm {mm}^{2}$ of surface area because this is the scale at which the intra-area connectivity is well described by the data sources (Potjans and Diesmann 2014). We can thus derive a multi-scale connectivity graph based on this local extent but not yet for entire areas where further spatial features such as patchy connections emerge. However, for completeness we do estimate the overall connectivity to each area arising outside the network, i.e., the combined external inputs from outside the $1\,\mathrm {mm}^{2}$ patches as well as from cortical and subcortical regions not included in the model. These external inputs are relevant for possible future extensions and for dynamical simulations of the system, complementing the population sizes and the internal connectivity map. Figure 1 provides an overview of the derivation of the network model.

Each area contains an excitatory (E) and an inhibitory (I) population in each of the layers 2/3, 4, 5 and 6 (L2/3, L4, L5, L6), except for area TH, which lacks L4. Neurons in the network receive inputs from four different sources: synapses from within the $1\,\mathrm {mm}^{2}$ patches, intra-area synapses from outside the $1\,\mathrm {mm}^{2}$ patches, cortico-cortical synapses from other areas in the network, and synapses from subcortical regions and cortical areas not included in the network. We refer to these four types of synapses, respectively, with the Roman numerals I–IV. In the following, we detail the data sources used to derive the network structure of the model, that is, the population sizes, the local and cortico-cortical (inter-area) connectivity and the external input. Table 1 lists all data sources used in this study. Table 2 gives an overview of the heuristics used to derive the model in combination with the available experimental data. Table 3 summarizes all variables and parameters appearing in the calculations.

**Table 1:** Overview of the data sources used

Data modality	Sources
Layer-resolved neuronal volume densities	Personal communication, H. Barbas and C.-C. Hilgetag
Architectural types	Hilgetag et al. (2016, Table 4)
Total cortical thicknesses	Hilgetag et al. (2016, Table 4)
Laminar thicknesses, estimated from micrographs	O’Kusky and Colonnier (1982), Boussaoud et al. (1990), Rakic et al. (1991), Preuss and Goldman-Rakic (1991), Rockland (1992), Felleman et al. (1997), Petrides and Pandya (1999), Angelucci et al. (2002), Lavenex et al. (2002), Suzuki and Amaral (2003), Rozzi et al. (2006), Eggan and Lewis (2007), Markov et al. (2014a)
Ratios of excitatory to inhibitory cell counts	Binzegger et al. (2004)
Surface areas	Computed with Caret (Van Essen et al. 2001) on the basis of each area’s representation on the F99 cortical surface (Van Essen 2002)
Local microcircuit scheme	Potjans and Diesmann (2014, Table 5), largest contributions from Binzegger et al. (2004), Thomson and Lamy (2007)
Intrinsic fractions of labeled neurons ($\hbox {FLN}_{\mathrm {i}}$)	Markov et al. (2011)
Average number of synapses per receiving neuron (indegree) in monkey V1	Cragg (1967), O’Kusky and Colonnier (1982)
Binary connectivity matrix for cortico-cortical connections	Stephan et al. (2001), Bakker et al. (2012), Suzuki and Amaral (1994a), Felleman and Van Essen (1991), Rockland and Pandya (1979), Barnes and Pandya (1992)
Fractions of labeled neurons (FLN)	Markov et al. (2014a)
Fractions of supragranular labeled neurons (SLN)	Markov et al. (2014b)
Laminar source patterns of cortico-cortical connections from retrograde tracing	Felleman and Van Essen (1991), Barnes and Pandya (1992), Suzuki and Amaral (1994b), Morel and Bullier (1990), Perkel et al. (1986), Seltzer and Pandya (1994)
Laminar target patterns of cortico-cortical connections from anterograde tracing	Jones et al. (1978), Rockland and Pandya (1979), Morel and Bullier (1990), Webster et al. (1991), Felleman and Van Essen (1991), Barnes and Pandya (1992), Distler et al. (1993), Suzuki and Amaral (1994b), Webster et al. (1994)
Statistical relations between synapse and cell body locations in cat V1	Binzegger et al. (2004)

### Population sizes

We estimate the number of neurons in each area and population in three steps:

1. Layer-resolved neuronal volume densities for 14 areas were provided by H. Barbas (personal communication; for details see Supplementary Sec. “Neuron densities”). We translate the neuronal densities to the FV91 scheme from the most representative area in the original scheme (Supplementary Table S2). Architectural types reflect the distinctiveness of the lamination as well as L4 thickness, with agranular cortices having the lowest and V1 the highest value. Table 4 of Hilgetag et al. (2016) lists the architectural types, which we translate to the FV91 scheme according to Supplementary Table S2. To the previously unclassified areas MIP and MDP we manually assign type 5 matching their neighboring area PO, which is similarly involved in visual reaching (Johnson et al. 1996; Galletti et al. 2003), and was placed at the same hierarchical level by Felleman and Van Essen (1991). For areas not covered by the data set, we take the average laminar densities for areas of the same architectural type.
2. Total cortical thicknesses are given in Hilgetag et al. (2016, Table 4) for the same areas for which neuron densities were measured. Missing values are filled in using a linear fit of total thickness versus logarithmized overall neuron density, which reflects architectonic differentiation similarly to architectural types, but has the advantage of being continuous (Beul et al. 2017). Quantitative data from the literature combined with our own estimates from published micrographs (Supplementary Table S3) determine relative laminar thicknesses.
3. The fraction of excitatory neurons in each layer is taken to be identical across areas. For the laminar dependency, values from cat V1 (Binzegger et al. 2004) are used with 78% excitatory neurons in L2/3, 80% in L4, 82% in L5, and 83% in L6.

### Local connectivity

The connection probabilities of the microcircuit model (Potjans and Diesmann 2014, Table 5) form the basis for the local circuit of each area. They provide an $8\times 8$ matrix of population-specific connection probabilities that was compiled from anatomical and electrophysiological studies (with large contributions from Binzegger et al. 2004; Thomson and Lamy 2007). We adapt this circuit to all 32 areas by preserving the relative indegrees between local projections which leads to area-specific connection probabilities. To determine the fraction of type I and II (i.e., within-area) synapses for each area, we use retrograde tracing data from Markov et al. (2011) consisting of fractions of labeled neurons (FLN) per area as a result of injections into one area at a time. The measured FLN thus determine the numbers of source neurons for each projection. The fraction intrinsic to the injected area, $\hbox {FLN}_{\mathrm {i}}$, is approximately equal for all nine areas where this fraction was determined, with a mean of 0.79. We assume that source neurons on average establish the same number of synapses in a given target area, independent of their location (inside or outside the given area). Combining this with the area-specific total numbers of synapses leads us to the total numbers of local synapses, which we distribute as further detailed in the “Results” section.

### Cortico-cortical connectivity

We treat all cortico-cortical connections as originating and terminating in the $1\,\mathrm {mm}^{2}$ patches, ignoring their spatial divergence and convergence. We determine whether a pair of areas is connected based on the union of all connections reported in the FV91 scheme in the CoCoMac database (Stephan et al. 2001; Bakker et al. 2012; Suzuki and Amaral 1994a; Felleman and Van Essen 1991; Rockland and Pandya 1979; Barnes and Pandya 1992) (see Supplementary Sec. “Processing of CoCoMac data” for details) and all connections reported by Markov et al. (2014a). Numbers of synapses between areas are determined on the basis of the retrograde tracing data from Markov et al. (2014a). The data consist of fractions of labeled neurons $\hbox {FLN}_{AB}=\hbox {NLN}_{AB}/\sum _{B^{\prime }}\hbox {NLN}_{AB^{\prime }}$ (analogous to the intrinsic fraction of labeled neurons $\hbox {FLN}_{\mathrm {i}}$), with $\hbox {NLN}_{AB}$ the number of labeled neurons in area *B* upon injection in area *A*. To translate the data into numbers of synapses, we assume, similarly to the assumption on intrinsically versus extrinsically labeled neurons, that a neuron projecting to a target area establishes the same number of synapses regardless of the source area it is located in. Markov et al. (2014a) used a parcellation scheme called M132 which is also available as a cortical surface, both in native and in F99 space, a standard macaque cortical surface included with Caret (Van Essen et al. 2001). For each injection, we identify the corresponding area in the FV91 parcellation (Supplementary Table S4) by registering the coordinates of the injection site to the F99 atlas available via the Scalable Brain Atlas (Bakker et al. 2015). There are data for 11 visual areas in the FV91 scheme with repeat injections in six areas, for which we take the arithmetic mean. To map data on the source side from M132 to FV91, we count the number of overlapping triangles on the F99 surface between any given pair of regions and distribute the FLN proportionally to the amount of overlap, using the F99 region overlap tool at the CoCoMac site (http://cocomac.g-node.org). To fill in gaps in the FLN data, we exploit the exponential decay of connection density with inter-areal distance (Ercsey-Ravasz et al. 2013). Supplementary Table S5 lists all distance values, which we compute as the median of the distances between all vertex pairs of the two areas in their surface representation in F99 space.

Layer-specific tracing results from the CoCoMac database (Stephan et al. 2001; Bakker et al. 2012) and Markov et al. 2014b help us determine the distribution of connections across source and target layers. On the source side, the laminar projection pattern can be expressed as the fraction of supragranular labeled neurons (SLN) in retrograde tracing experiments (Markov et al. 2014b). To map the SLN from the M132 to the FV91 scheme, we use the exact coordinates of the injections to determine the corresponding target area *A* in the FV91 parcellation, and for each pair of areas we take the mean SLN across injections. At this point, the source areas are still in the M132 parcellation. To map the source areas from M132 to FV91, we weight the SLN by the overlap $c_{B,\beta }$ between area $\beta$ in the former (M132) and area *B* in the latter (FV91) scheme and the FLN,

$$\begin{aligned} \hbox {SLN}_{AB}=\frac{\sum _{\beta }c_{B,\beta }\hbox {FLN}_{A,\beta }\hbox {SLN}_{A,\beta }}{\sum _{\beta }c_{B,\beta }\hbox {FLN}_{A,\beta }}. \end{aligned}$$

This weighting with the FLN reflects the fact that denser connections more strongly determine the overall distribution of labeled neurons across supra- and infragranular layers. We estimate missing values based on a sigmoidal fit of SLN versus the logarithmized ratio of overall cell densities of the two areas (Fig. 5a). This is similar to the relation between SLN and the hierarchical level differences found by Markov et al. (2014b), although there, the hierarchical ordering of areas was obtained using the SLN data in the first place. With this approach, the goodness of fit is difficult to evaluate, because some degrees of freedom are used up to determine the hierarchy itself. A relationship between laminar patterns and log ratios of neuron densities was suggested by Beul et al. (2017). Following Markov et al. (2014b), we use a beta-binomial model, assuming the numbers of labeled neurons in the source areas to sample from a beta-binomial distribution (e.g., Weisstein 2005). This distribution arises as a combination of a binomial distribution with probability *p* of supragranular labeling in a given area, and a beta distribution of *p* across areas with dispersion parameter $\phi$. With the probit link function *g* (e.g. McCulloch et al. 2008), the measured $\hbox {SLN}_{AB}$ relates to the log ratio $\ell _{AB}$ of overall neuron densities for each pair of areas as

1 $$\begin{aligned} g(\hbox {SLN}_{AB})=a_{0}+a_{1}\ell _{AB}, \end{aligned}$$

where $\{a_{0},a_{1}\}$ are scalar fit parameters. We perform this fit in the original scheme (M132) under the assumption that mapping cell densities between schemes introduces fewer errors than mapping SLN would. For mapping the cell densities to M132 we again employ the overlap tool of CoCoMac (see above) and compute the cell density of each area in the M132 scheme as a weighted average over the associated FV91 areas. For areas with identical names in both schemes, we simply take the neuron density from the FV91 scheme. We compute the SLN fit in R (R Core Team 2015) with the betabin function of the aod package (Lesnoff and Lancelot 2012). In contrast to Markov et al. (2014b), who exclude certain areas when fitting SLN versus hierarchical distance in view of ambiguous hierarchical relations, we take all data points into account to obtain a simple and uniform rule. We also tested a logit link function and found nearly identical results (Supplementary Fig. S1C).

As a further step, we combine SLN with tracing results from CoCoMac (Felleman and Van Essen 1991; Barnes and Pandya 1992; Suzuki and Amaral 1994b; Morel and Bullier 1990; Perkel et al. 1986; Seltzer and Pandya 1994). The data sets complement each other: SLN provides quantitative information on laminar patterns of outgoing projections for about one quarter of the connected areas, distinguishing only between supra- and infragranular layers. CoCoMac has values for all six layers (which we denote by $\alpha (\nu )$), but limited to a qualitative strength ranging from 0 (absent) to 3 (strong) which we take to represent numbers of synapses in orders of magnitude (for further details see Supplementary Sec. “Processing of CoCoMac data”). On the target side, we determine the pattern of target layers $P_{\mathrm {t}}$ from anterograde tracer studies in CoCoMac (Jones et al. 1978; Rockland and Pandya 1979; Morel and Bullier 1990; Webster et al. 1991; Felleman and Van Essen 1991; Barnes and Pandya 1992; Distler et al. 1993; Suzuki and Amaral 1994b; Webster et al. 1994) if available (29% coverage), and otherwise determine it from the source pattern, as further described in the “Results” section.

Anterograde tracing experiments characterize target patterns of projections in terms of the locations of the synapses, whereas the layer that forwards incoming input depends on the location of the cell body. Therefore, to characterize polysynaptic pathways, it is necessary to bridge the descriptions in terms of cell body and synapse locations. To this end, we relate synapse to target cell body locations using the following cat V1 data from Binzegger et al. (2004), which are listed in the table in Fig. 9 of Izhikevich and Edelman (2008): first, the probability $\mathcal {P}(s_{\mathrm {cc}}|c_{\mathrm {B}}\bigcap s\in v)$ for a synapse in layer *v* on a cell of type $c_{\mathrm {B}}$ (e.g., a pyramidal cell with soma in L5) to be of cortico-cortical origin (20th column in the table in Fig. 9 of Izhikevich and Edelman 2008); second, the relative occurrence ${\mathcal {P}}(c_{\mathrm {B}})$ of the cell type $c_{\mathrm {B}}$ (first column); and third, the total numbers of synapses $N^{\mathrm {syn}}(v,c_{\mathrm {B}})$ in layer *v* onto individual cells of the given type (second column). The latter do not equal the numbers of synapses onto the neurons in our network; we rather use them as auxiliary quantities in the calculation. Binzegger et al. (2004) distinguish 17 different cell types, which we map to the 8 cortical populations considered in our network based on the laminar position of their cell body and their excitatory or inhibitory nature (Supplementary Table S10). To transform the data of Binzegger et al. (2004) from cat to macaque, we adjust the occurrence of each cell type $c_{\mathrm {B}}$ associated with a population *i* according to the different relative population sizes in cat and macaque V1. For this, we compute the occurrence of population *i* in macaque V1, $\mathcal {P}_{i,\mathrm {V1}}=N_{i,\mathrm {V1}}/N_{\mathrm {total,}V1}$ and divide it by the sum of occurrences of all cell types associated with population *i* in cat. The occurrence of $c_{\mathrm {B}}$ is then multiplied by this factor: ${\mathcal {P}}(c_{\mathrm {B}})\rightarrow \mathcal {\mathcal {\mathcal {P}}}(c_{\mathrm {B}})\cdot \mathcal {P}_{i,\mathrm {V1}}/\sum _{c_{\mathrm {B}}^{\prime }\in i}\mathcal {P}(c_{B}^{\prime })$. The “Results” section details the corresponding derivation.

Cortico-cortical feedback connections preferentially target excitatory rather than inhibitory neurons, i.e., a disproportionately high number of synapses is formed onto excitatory neurons (Johnson and Burkhalter 1996; Anderson et al. 2011). We choose a fraction of 93% of connections targeting excitatory neurons, as an average over experimental values ranging between 87 and 98%.

### External input

Intra-areal synapses originating outside the $1\,\mathrm {mm}^{2}$ patches (type II) and those coming from outside vision-related cortex, that is, non-visual and subcortical inputs (type IV) are external inputs for our purposes. While they do not form an intrinsic part of the system under consideration, these inputs provide a more comprehensive picture of the network and are relevant for investigations of the network dynamics. Therefore, we estimate these inputs for completeness. As further explained in the results, we can estimate the numbers of type II synapses to some extent from local connectivity profiles, but the available data do not allow us to faithfully determine the contribution from remote intra-area connectivity (patchy connections) for all areas. Furthermore, quantitative area-specific data on non-visual and subcortical inputs are highly incomplete. For these reasons, we jointly describe the type II and type IV synapses using a simple scheme: for each area, we compute the total number of external synapses as the difference between the total number of synapses (determined from the volume density of synapses) and those of type I and III and distribute these such that all neurons in the given area have the same indegree for external sources. Supplementary Table S6 lists the resulting external indegrees. Overall, external inputs amount to approximately 32% of the total inputs to each neuron in the network.

**Table 2:** Table of the heuristics and regularities used to construct the model along with starting points for extensions, if applicable

Feature	Heuristic	Argument	Starting points for extensions
Population sizes	Neuron densities of areas with missing data equal the mean neuron density for areas of the same architectural type.	Neuron density varies systematically with architectural type	
Population sizes	Areas MIP and MDP have architectural type 5	Their neighboring area PO, similarly involved in visual reaching (Johnson et al. 1996; Galletti et al. 2003), is of type 5 (Hilgetag et al. 2016)	
Population sizes	Total thickness and relative laminar thicknesses for areas with missing data are linearly predicted from the logarithm of their overall neuron density	This follows observed gradients. The increase in relative L4 thickness with log neuron density is consistent with L4 thickness entering into the definition of the architectural types	
Population sizes	The fraction of excitatory neurons in each layer is identical across areas	This provides a simple rule across areas, for lack of systematic area-specific data	Beaulieu et al. (1992) report similar values for layer-specific fractions of inhibitory neurons in macaque V1. Gabbott and Bacon (1996) report layer-specific fractions of inhibitory neurons in macaque medial prefrontal cortex differing from the values of Beaulieu et al. (1992)
Local connectivity	We assume an underlying Gaussian model for the local connection probability	This ansatz provides consistency with the derivations of Potjans and Diesmann (2014)	Markov et al. (2011) report an exponential decay of locally labeled neurons with distance from the injection site. With assumptions on cell density, this enables deriving a non-Gaussian distance-dependent connection probability
Local connectivity	Population pairs have the same relative indegrees as in the model of Potjans and Diesmann (2014)	This follows the notion of a canonical microcircuit (Douglas et al. 1989; Douglas and Martin 2004), for lack of comprehensive species- and area-specific data	Beul and Hilgetag (2015) suggest a canonical microcircuit for agranular cortical areas, which in our model includes area TH
Local connectivity	The relative amount of local synapses is constant across areas	The fraction of labeled neurons intrinsic to the injected area found by retrograde tracing is approximately constant	
Long-range connectivity	All cortico-cortical connections originate and terminate in the $1\,\mathrm {mm}^{2}$ patches covered by our model	Since we do not explicitly include spatial dependence of connections, we opt for a simple model for cortico-cortical connections	Cortico-cortical connections exhibit divergence and convergence (Colby et al. 1988; Salin et al. 1989; Gattass et al. 1997; Markov et al. 2014b)
Long-range connectivity	All cortico-cortical connections are excitatory	This simplification approximates the finding that the large majority of cortico-cortical projections are excitatory	A small fraction of cortico-cortical connections in monkey (Tomioka and Rockland 2007) and other species (McDonald and Burkhalter 1993; Gonchar et al. 1995; Fabri and Manzoni 1996, 2004; Tomioka et al. 2005; Pinto et al. 2006; Higo et al. 2007) are inhibitory
Long-range connectivity	Neurons in all source areas form the same number of synapses in each target area	This assumption allows us to directly translate FLN into synapse numbers	There is evidence that numbers of cortico-cortical synapses per neuron differ between feedback and feedforward connections (Rockland 2003)
Long-range connectivity	The probability for a postsynaptic neuron to form a cortico-cortical synapse in a specific layer is constant across areas.	For lack of data in areas besides V1, we take the computed values from the Binzegger et al. (2004) data as representative across the model	
Long-range connectivity	The probability for a synapse to be established on a neuron of a given type is proportional to the length of the dendrites of the neuron type in the given layer	This heuristic is a version of Peters’s rule, which has been shown to have reasonably wide validity at the population level (Rees et al. 2016)	
Long-range connectivity	The relative number of synapses sent by supragranular neurons is filled in based on the logarithmic ratio of overall cell densities in the two participating areas	This follows the observed relation between SLN and the log ratio of overall cell densities in combination with interpreting ratios of labeled neurons as ratios of formed synapses	
Long-range connectivity	The level of SLN predicts the type of laminar termination pattern	This follows the observed relation between SLN and termination pattern	
Long-range connectivity	Feedforward and feedback pathways are not separate within layers: individual neurons can send both types of connections	This heuristic is used to avoid the added complexity that would result from further subdivisions of the neural populations	A finer definition of laminar pathways may be achieved via a dual counterstream organization (Markov et al. 2014b)

### Analysis methods

We investigate the community structure of the area-level network with the map equation method (Rosvall et al. 2009). In this clustering algorithm, an agent performs random walks between graph nodes according to their degree of connectivity and a certain probability of jumping to a random network node. We choose the probability for a certain target node to be selected to be proportional to the outdegree of the connection, and $p=0.15$ as the probability of a random jump. The algorithm detects clusters in the graph by minimizing the length of a binary description of the network using a Huffman code. To assess the quality of the clustering, we compute a modularity measure which extends a measure for unweighted, directed networks (Leicht and Newman 2008) to weighted networks, analogous to Newman (2004),

$$\begin{aligned} Q=\frac{1}{m}\sum _{A,B}\left( \mathcal {K}_{AB}^{\mathrm {out}}-\frac{\sum _{B^{\prime }}\mathcal {K}_{AB^{\prime }}^{\mathrm {out}}\cdot \sum _{A^{\prime }}\mathcal {K}_{A^{\prime }B}}{m}\right) \delta _{\mathcal {C}_{A},\mathcal {C}_{B}}, \end{aligned}$$

where $\mathcal {K}_{AB}^{\mathrm {out}}$ and $\mathcal {K}_{AB}$ respectively are the matrices of relative outdegrees and indegrees, $m=\sum _{A,B}\mathcal {K}_{AB}^{\mathrm {out}}$ and $\delta _{\mathcal {C}_{A},\mathcal {C}_{B}}=1$ if areas *A* and *B* are in the same cluster and 0 otherwise. $Q=0$ reflects equal connectivity within and between clusters, while $Q=1$ corresponds to connectivity exclusively within clusters.

To study paths in the network, we construct the weighted and directed graph of the network connectivity at the population level. While this graph only contains anatomical information, to identify the paths that are most relevant for activity propagation we take into account (1) the relative weight of inhibitory compared to excitatory synapses; and (2) the near-criticality of the brain (Robinson et al. 2014; Priesemann et al. 2014). Following Potjans and Diesmann (2014), we define the synaptic weight $J_{\mathcal {E}}=0.15\,\mathrm {mV}$ for excitatory connections and $J_{\mathcal {I}}=- \,4J_{\mathcal {E}}$ for inhibitory connections. We then construct a weight matrix *G* with elements $g_{ij}=K_{ij}\cdot |J|$ where *J* is chosen depending on whether the source population is excitatory or inhibitory. This matrix is transformed into an approximate gain matrix by scaling the matrix by a factor representing the susceptibility of the target populations, i.e., the change in output activity for a unit change in input. For simplicity, we assume this susceptibility to be identical across populations. To reflect the near-criticality of the brain, we choose it to be equal the reciprocal of the maximal real part of the eigenvalues of *G*: $G^{\prime }=G/\left[ {\mathrm {Re}}(\lambda )\right] _{\mathrm {max}}$, so that the maximal real part of the eigenvalues of the resulting matrix is $\left[ {\mathrm {Re}}(\lambda ^{\prime })\right] _{\mathrm {max}}=1$. This scaling is relevant because it modulates the relative strengths of direct and indirect paths: a larger value of $\left[ {\mathrm {Re}}(\lambda ^{\prime })\right] _{\mathrm {max}}$ increases the relative weighting of indirect paths. The weight of the edge from population *j* to *i* is then defined as $g_{ij}^{\prime }$. The distance between two nodes in the graph is defined as the logarithm of the reciprocal of the weight, $d_{ij}={\mathrm {log}}(1/w_{ij})$, so that summing the distances reflects a multiplication of the corresponding weights. We find the shortest paths between any two nodes of the graph using the Bellman–Ford algorithm (Shimbel 1955; Ford 1956; Bellman 1958). This algorithm finds the shortest paths emanating from vertex *i* on a graph with *N* vertices in an iterative manner: it starts by assigning an infinite path length to all other nodes *k* of the graph. Then, it loops through all edges (*j*, *k*) of the graph, tests if the path length $p_{ij}$ plus the distance of the edge $d_{jk}$ is smaller than the currently stored path length $p_{ik}$, and, if so, assigns $p_{ik}\rightarrow p_{ij}+d_{jk}$. By repeating this $N-1$ times for all edges, the algorithm considers paths of increasing length at every iteration and ultimately finds the shortest paths between each pair of vertices. In contrast to Dijkstra’s algorithm, it can deal with edges with negative distance values.

**Table 3:** Variable and parameter definitions

Variable	Explanation
*A*, *B*	Area
*i*, *j*	Population
*v*	Layer
${\mathcal {E}}$	Pool of excitatory neurons
${\mathcal {I}}$	Pool of inhibitory neurons
*S*	Surface area
*D*	Cortical thickness
*R*	Radius of a cortical area
$R_{0}$	Radius of a $1\,\mathrm {mm}^{2}$ area
*N*	Number of neurons
$\gamma$	Fraction of excitatory neurons
$\rho$	Volume density of neurons
$N_{\mathrm {syn}}$	Number of synapses
$\rho _{\mathrm {syn}}$	Volume density of synapses
$\sigma$	Spatial width of Gaussian profile underlying the intrinsic connectivity
$C_{0}$	Peak of Gaussian connectivity profile averaged across population pairs
$\overline{C}$	Connection probability averaged over all possible positions of two neurons
*K*($K^{\mathrm {out}}$)	Average indegree (outdegree) (number of synapses per target/source neuron)
$\mathcal {K}$($\mathcal {K}^{\mathrm {out}}$)	Relative average indegree (outdegree)
$c_{A}$	Area-specific conversion factor for indegrees
NLN	Number of labeled neurons (as in Markov et al. 2011)
FLN	Fraction of labeled neurons (as in Markov et al. 2011)
SLN	Fraction of supragranularly labeled neurons (as in Markov et al. 2014b)
*c*	Normalization constant of the decay of FLN over inter-area distance (see Eq. 10)
$\lambda$	Length constant of the decay of FLN over inter-area distance (see Eq. 10)
$d_{AB}$	Distance between areas *A* and *B* (see Eq. 10)
$c_{B,\beta }$	Overlap of area $\beta$ in the M132 scheme and area *B* in the FV91 scheme
$\phi$	Dispersion parameter of the beta-binomial distribution governing the labeling of neurons in source areas
$\ell$	Log ratio of neuron densities of two areas (see Eq. 1)
$a_{0},a_{1}$	Fit parameters of the sigmoidal SLN relation (see Eq. 1)
$c_{\mathrm {B}}$	Cell body
$s_{\mathrm {cc}}$	Cortico-cortical synapse
*S*	Pool of supragranular layers (i.e., layer 2/3)
*I*	Pool of infragranular layers (i.e., layers 5 and 6)
$P_{\mathrm {s}}$	Pattern of source layers
$P_{\mathrm {t}}$	Pattern of target layers
$\alpha (v)$	Qualitative connection strength for layer *v* from CoCoMac (see Supplementary Eq. 3)
$X_{j}$	Fraction of synapses formed by neurons in source population *j* (see Supplementary Eq. 3)
$Y_{v}$	Fraction of synapses formed in target layer *v* (see Supplementary Eq. 3)
$Z_{i}$	Factor for redistributing synapses to ensure the E–I specificity of cortico-cortical connections (see Supplementary Eq. 3)

## Results

In the following sections, we describe the definition of the network structure. Our goal is to derive the probability $C_{iA,jB}$ for two neurons in populations $i,\,j$ of areas $A,\,B$ in the network to be directly connected by one or more synapses. Each area is modeled as the volume under $1\,{\mathrm {mm}}^{2}$ of cortical surface, since local connectivity at this scale has been well characterized, whereas information about medium-range connectivity within areas is highly incomplete. The neurons of a specific cell type, excitatory (E) or inhibitory (I), in a particular area and layer (2/3, 4, 5 or 6) form a population in our network. For each pair of populations, we assume a uniform connection probability between neurons. Assuming that synapses between two populations are randomly distributed, allowing for multiple contacts between neurons, the probability of at least one synapse between two neurons is (Potjans and Diesmann 2014, Eq. 1)

2 $$\begin{aligned} C_{iA,jB}=1-\left( 1-\frac{1}{N_{iA}N_{jB}}\right) ^{N_{iA,jB}^{\mathrm {syn}}}\,. \end{aligned}$$

For small connection probabilities, this reduces approximately to the number of synapses divided by the sizes of the source and target populations. To determine the connection probabilities in the network from Eq. (2), we thus need to know the population sizes *N* and the number of synapses $N^{\mathrm {syn}}$ between any pair of populations. In the following, we make use of the concept of average indegree, which is defined as the average number of synapses per receiving neuron,

3 $$\begin{aligned} K_{iA,jB}=\frac{N_{iA,jB}^{\mathrm {syn}}}{N_{iA}}. \end{aligned}$$

Henceforth, we refer to the average indegree for a pair of populations also simply as ‘indegree’. Outdegrees (also as average) are defined analogously as

4 $$\begin{aligned} K_{iA,jB}^{\mathrm {out}}=\frac{N_{iA,jB}^{\mathrm {syn}}}{N_{jB}}. \end{aligned}$$

Figure 1 provides an overview of the derivation of the network structure.

### Area-specific laminar compositions

**Fig. 2:**
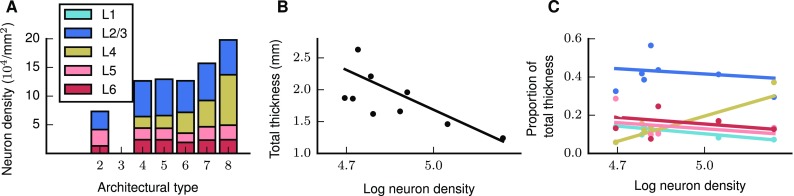
Aspects of cortical architecture determining population sizes. **a** Laminar neuron densities for the architectural types in the model. Type 2, here corresponding only to area TH, lacks L4. We treat L1 as containing synapses but no neurons. Data provided by H. Barbas (personal communication). **b** Total thickness versus logarithmized overall neuron density and linear least-squares fit ($r=-\,0.7,\;p=0.005$). **c** Relative laminar thickness (see Supplementary Table S3) versus logarithmized overall neuron density and linear least-squares fits (L1: $r=-\,0.51,\;p=0.08$, L2/3: $r=-\,0.20,\;p=0.52$, L4: $r=0.89,\;p=0.0001$; L5: $r=-\,0.31,\;p=0.36$, L6: $r=-\,0.26,\;p=0.43$). Total cortical thicknesses *D*(*A*) and overall neuron densities for 14 areas from Hilgetag et al. (2016), Table 4. The overall densities are based on Nissl staining for 11 areas and for 3 areas on NeuN staining. Laminar neuron densities are based on NeuN staining for all 14 areas. Values based on NeuN staining are linearly scaled to account for a systematic undersampling as determined by repeat measurements in the 11 aforementioned areas

We here derive the number of neurons in each population of the network from measured and estimated neuron densities, laminar thicknesses, and proportions of excitatory and inhibitory neurons. Overall neuron density and L4 neuron density increase with architectural type (Fig. 2a). Since we assign neuron densities to areas with missing data according to their architectural types, the same trends are present throughout the model. Total cortical thickness decreases with increasing logarithmized overall neuron density, $\mathrm {log}\,\rho$, providing thickness estimates for the 18 areas not included in the empirical data set (Fig. 2b). The ratio of L4 thickness and total cortical thickness increases with $\mathrm {log}\,\rho$, which predicts the relative L4 thickness for areas with missing data (Fig. 2c). Since the relative thicknesses of the other layers show no notable change with $\mathrm {log}\,\rho$, we fill in missing values using the mean of the known data for these quantities and then normalize the sum of the relative thicknesses to 1 (Supplementary Table S7).

For deriving the local connectivity, as an intermediate step we require the full surface areas (Supplementary Table S8) to sample the tails of the Gaussian connectivity profiles, and not just the $1\,\mathrm {mm}^{2}$ patches. For this purpose, we approximate each brain area as a flat disk with radius *R* and surface area $S(R)=\pi R^{2}$, so that the number of neurons in population *i* of area *A* is

5 $$\begin{aligned} N_{iA}(R)=\rho _{A,v_{i}}S(R)D_{A,v_{i}} \cdot &{\left\{\!\begin{array}{ll} \gamma _{v_{i}} &{}\quad {\text {if }}\;i\in \mathcal {E}\\ 1-\gamma _{v_{i}} &{}\quad {\text {if }}\;i\in \mathcal {I} \end{array}\right. }&, \end{aligned}$$

where $v_{i}$ denotes the layer of population *i*, $D_{A,v_{i}}$ the thickness of layer $v_{i}$, and $\mathcal {E},\,\mathcal {I}$ the pool of excitatory and inhibitory populations, respectively. Supplementary Table S9 gives the population sizes corresponding to the $1\,\mathrm {mm}^{2}$ area size we consider.

### A comprehensive picture of network connectivity

**Fig. 3:**
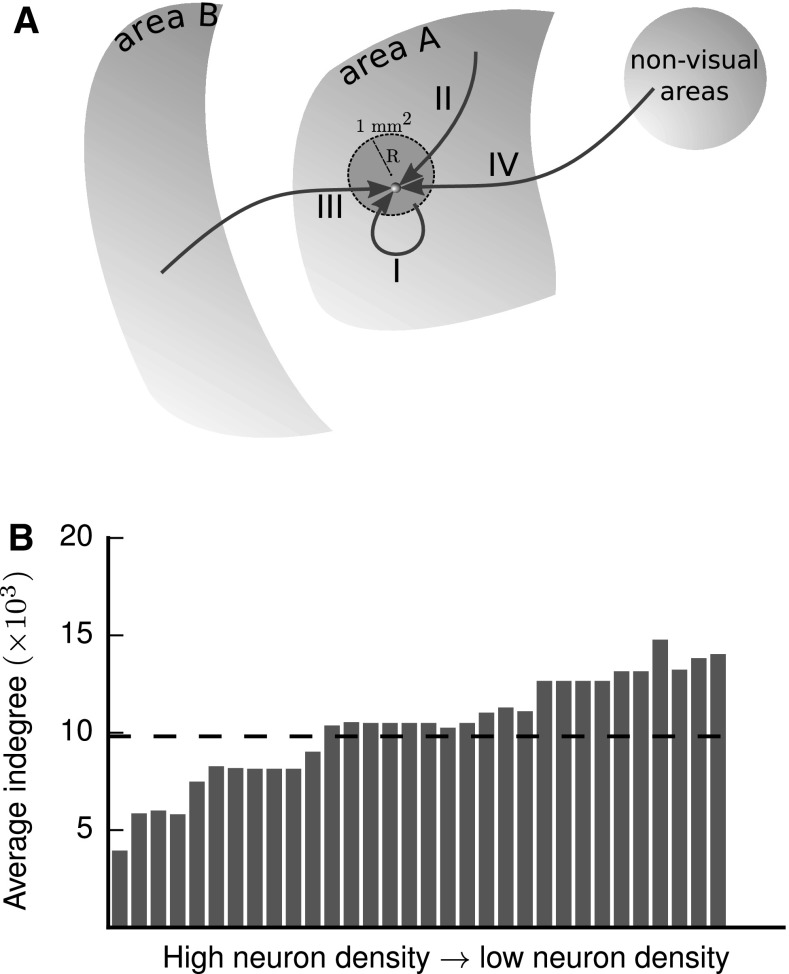
Construction principles of the network connectivity. **a** Each neuron receives four different types of connections. I: Intra-area synapses from within the $1\,\mathrm {mm}^{2}$ patch, II: Intra-area synapses from outside the $1\,\mathrm {mm}^{2}$ patch, III: Cortico-cortical synapses from vision-related areas, IV: Synapses from subcortical and non-visual cortical areas. **b** Average number of synapses per neuron across the 32 areas of the network versus overall neuron density. The dashed line shows the average indegree across all neurons of the network

Each neuron receives synapses of four different origins (Fig. 3a): those originating inside the $1\,\mathrm {mm}^{2}$ microcircuit (type I), the remaining intra-areal synapses (type II), cortico-cortical synapses from other vision-related areas (type III), and synapses from outside vision-related cortex (type IV).

For combined local and long-range connections, we assume a constant volume density of synapses across areas (Harrison et al. 2002). Experimental values for the average indegree in monkey V1 vary between 2300 (O’Kusky and Colonnier 1982) and 5600 (Cragg 1967) synapses per neuron. We take the average (3950) as representative for V1, resulting in a synapse density of $\rho _{\mathrm {syn}}=8.3\times 10^{8}\;\frac{\text {synapses}}{\text {mm}^{3}}$, not far from the value of $6.3\times 10^{8}\;\frac{\text {synapses}}{\text {mm}^{3}}$ measured in rat somatosensory cortex (Markram et al. 2015). This constant synapse density diversifies the areas in terms of their connectivity due to their different neuron densities. Combined with decreasing cell density along the gradient of architectural types (Fig. 2a), the constant synapse density leads to an increase in the average indegree of neurons in low-type areas compared to high-type areas (Fig. 3b). Primary visual cortex V1 has the lowest average indegree of $\sim$3950 synapses per neuron while neurons in the area with the lowest architectural type, TH, receive on average $\sim$14,000 synapses. The average indegree does not strictly increase with the overall neuron density due to differences in the area-specific laminar composition. Averaged across all areas, a neuron in the network receives approximately 9800 synapses.

We base the fraction of intra-areal synapses (types I + II) on fractions of labeled neurons intrinsic to the injected area ($\hbox {FLN}_{\mathrm {i}}$) in retrograde tracing experiments by Markov et al. (2011). Since the reported values are approximately constant across injected areas, we use the mean value of 0.79 for all 32 areas of the network. This leads us to the assumption that 79% of the synapses to a neuron are intra-areal (types I and II combined) and the remaining 21% stem from sources outside of the areas (types III and IV combined).

In the following two subsections, we explain the integration of the different data sources to yield the area- and population-specific numbers of synapses of types I and III.

#### Scalable scheme of local connectivity

Our network structure does not include distance dependence of connections within each population. However, to distinguish between synapses of type I and II, we take the underlying probability *C* for a given neuron pair to establish one or more contacts to decay with distance according to a Gaussian with standard deviation $\sigma =297$ μm (Potjans and Diesmann 2014). We approximate each brain area as a flat disk with radius *R*. The radius determines the cut-off of the Gaussian and hence lets us determine the ratio between the numbers of type I and type II synapses. The average connection probability is obtained by integrating over all possible positions of the two neurons (cf. Supplementary Eq. 1). Averaged across population pairs in cat V1, $C_{0}$ is 0.143 (computed from Eq. 8 and Table S1 in Potjans and Diesmann 2014). Note that Potjans and Diesmann (2014) only vary the position of one neuron, keeping the other neuron fixed in the center of the disk (Eq. 9 in that paper). In adjusting the local connectivity to the area-specific surface areas in our model, we thus need to take into account the method for integrating the Gaussian profiles. Since mean synaptic inputs are proportional to the indegrees, we consider indegrees a defining characteristic of the local circuit. The model assumes that the relative indegrees between population pairs are like those in cat V1 adjusted for surface area and integration method. Thus, the different population sizes and cortical thicknesses in the macaque areas compared to cat V1 do not affect the relative indegrees across population pairs, but they are still relevant for the absolute numbers of synapses and thereby for the connection probabilities. Henceforth, we denote connection probabilities computed with the approach of Potjans and Diesmann (2014) with the subscript PD14 and use primes for all variables referring to a network with the cortical thickness and relative population sizes of the microcircuit model of Potjans and Diesmann (2014). The same variables without primes refer to the corresponding quantities in the macaque areas.

The parameters of the microcircuit model are reported for a $1\,\mathrm {mm}^{2}$ patch of cortex, corresponding to $R=\sqrt{1/\pi }\,\mathrm {mm}$, which we call $R_{0}$. For each source population *j* and target population *i*, we first translate the connection probabilities of the $1\,\mathrm {mm}^{2}$ model to a variable radius *R* via

6 $$\begin{aligned} C_{ij}^{\prime }(R)=C_{ij,\mathrm {PD}14}^{\mathrm {\prime }}\left( R_{0}\right) \frac{\bar{C}^{\prime }(R)}{\bar{C}_{\mathrm {PD}14}^{\prime }\left( R_{0}\right) }, \end{aligned}$$

with $\bar{C}_{\mathrm {PD}14}^{\prime }(R_{0})=0.066$. Equation (6) reflects the local connection probabilities as they would be in an area with surface area $\pi R^{2}$, taking into account all possible pairs of neuron positions rather than fixing one neuron in the center, but before adjustment for the area-specific population sizes and the total number of local synapses. To preserve relative indegrees, we set

$$\begin{aligned} \frac{K_{ij,A}(R)}{K_{kl,A}(R)}=\frac{K_{ij}^{\prime }(R)}{K_{kl}^{\prime }(R)}\quad \forall i,j,k,l, \end{aligned}$$

which is equivalent to scaling all indegrees by an area-specific conversion factor $c_{A}(R)$,

7 $$\begin{aligned} K_{ij,A}(R)=c_{A}(R)K_{ij}^{\mathrm {\prime }}(R)\quad \forall i,j. \end{aligned}$$

The conversion factor $c_{A}(R)$ is larger for areas with smaller neuron densities because of the assumption of a constant volume density of synapses. As explained in the Supplementary Sec. “Local connectivity”, it is computed as

8 $$\begin{aligned} c_{A}(R)=\frac{N_{A}^{\mathrm {syn,tot}}(R)}{\sum _{i,j}N_{iA}(R)K_{ij}^{\prime }}\;\; \hbox {FLN}_{\mathrm {i}}\Bigg \langle \frac{K_{ij}^{\prime }(R)}{K_{ij}^{\prime }(R_{\mathrm {full}})}\Bigg \rangle _{ij}\,, \end{aligned}$$

with $\hbox {FLN}_{\mathrm {i}}$ the fraction of labeled neurons intrinsic to the injected area (Markov et al. 2011) and $N^{\mathrm {syn,tot}}(R)=\rho _{\mathrm {syn}}\pi R^{2}D$ with *D* the total thickness of the given area and $R_{\mathrm {full}}$ the radius of a disk with full area-specific surface *S*(*A*) (Supplementary Table S8). Choosing $R=R_{0}$ in Eqs. (8) and (7) yields the numbers of local synapses:

9 $$\begin{aligned} N_{ij,A}^{\mathrm {syn,I}}=c_{A}(R_{0})K_{ij}^{\prime }(R_{0})N_{iA}(R_{0}). \end{aligned}$$

Modeling each area as a $1\,\mathrm {mm}^{2}$ patch leads to connections outside $R_{0}/\sigma =\sqrt{1/\pi }\,\mathrm {mm}/297\,\mathrm {\mu m}=1.9$ times the standard deviation of the Gaussian falling outside the patch. The corresponding inputs are treated as external input (type II synapses). To determine their total number for an area, we use Eq. (9) with $R=R_{\mathrm {full}}$, sum over all population pairs of the area, and subtract the total number of type I synapses.

Our assumptions lead to a scalable scheme of the local circuit over a continuous range of modeled sizes so that local type I synapses increase at the cost of external type II synapses. However, when going beyond the $1\,\mathrm {mm}^{2}$ scale, one would have to take into account patchy connectivity within areas, i.e., spatial clustering of remote intra-area connections, which would refine the trade-off of type I and type II synapses.

#### Layer-specific heterogeneous cortico-cortical connectivity

Population-specific numbers of modeled cortico-cortical synapses are determined in three steps: (1) deriving the area-level connectivity; (2) distributing synapses across layers; (3) assigning synapses to target neurons.

**Fig. 4:**
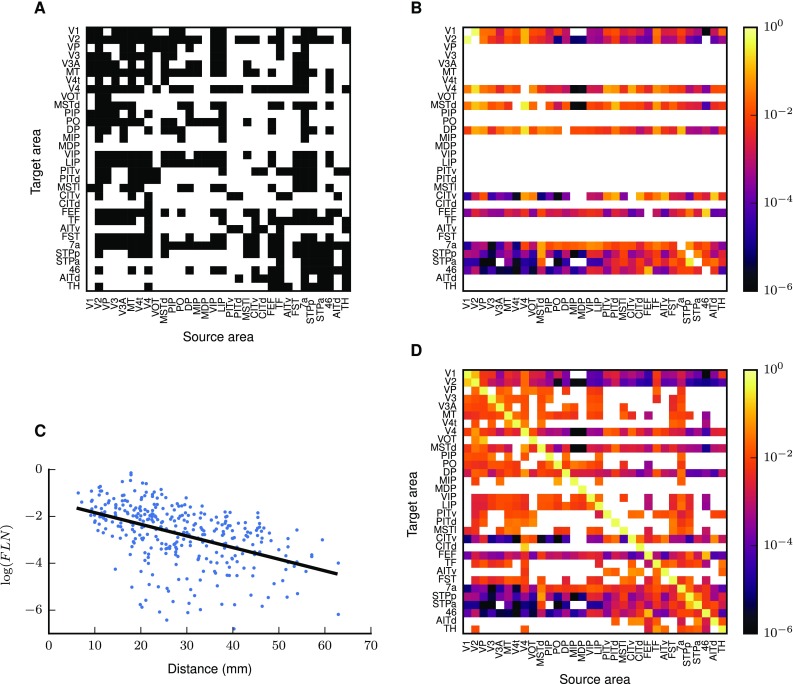
Combination of binary and quantitative tracing data into an area-level connectivity map. **a** Binary connectivity from CoCoMac. Black, existing connections; white, absent connections. **b** Fractions of labeled neurons (FLN) from Markov et al. (2014a) mapped from their parcellation scheme (M132) to that of Felleman and Van Essen (1991). **c** Connection densities decay exponentially with inter-area distance. Black line, linear regression with $\log (\hbox {FLN})=\left(\ln 10 \right)^{-1} \cdot \left( \ln c-\lambda d \right)$ ($c=0.045,\;\lambda =0.11\,\mathrm{mm}^{-1},\;p=10^{-19}$; cf. Eq. (10)). **d** Area-level connectivity of the model, based on data in **a**–**c**, expressed as relative indegrees for each target area

Two areas are connected if the connection is in the CoCoMac database (Stephan et al. 2001; Bakker et al. 2012) or was reported by Markov et al. (2014a). CoCoMac provides a binary connectivity matrix with a density of 45% (Fig. 4a). Markov et al. (2014a) quantitatively measured connection densities and found a number of previously unknown connections (Fig. 4b) leading to a total of 62% of all pairs of areas being connected. The data set of Markov et al. (2014a) consists of fractions of labeled neurons $\hbox {FLN}_{AB}$ in area *B* upon injection in area *A*. To estimate values for the areas not included in the data set, we use the exponential decay of cortico-cortical connectivity with distance between areas (Ercsey-Ravasz et al. 2013),

10 $$\begin{aligned} \hbox {FLN}_{AB}=c\cdot \exp \left( -\,\lambda d_{AB}\right) . \end{aligned}$$

A linear least-squares fit of the logarithm of the *FLN* yields a decay rate of $\lambda =0.11\,\mathrm {mm}^{-1}$ with high significance (Fig. 4c). The data of Markov et al. (2014a) expose an exponential distribution of axon lengths, independent of a parcellation of cortical space into areas. Analogously to Ercsey-Ravasz et al. (2013), we here employ this distribution as a descriptive model for the connection density between areas, which consequently depends on the parcellation scheme and potentially increases the variance of the data (see also Horvát et al. 2016). The total number of synapses $N_{{\mathrm {syn}},AB}$ between each pair of areas is assumed to be proportional to the number of labeled neurons $\hbox {NLN}_{AB}$ and thus to $\hbox {FLN}_{AB}$,

$$\begin{aligned} \frac{N_{\mathrm {syn},AB}}{\underbrace{\sum _{B^{\prime }}N_{\mathrm {syn},AB'}}_{=N_{\mathrm {syn,tot},A}}}=\frac{\hbox {NLN}_{AB}}{\sum _{B^{\prime }}\hbox {NLN}_{AB^{\prime }}}=\frac{\hbox {FLN}_{AB}}{\sum _{B'}\hbox {FLN}_{AB'}}. \end{aligned}$$

This corresponds to individual neurons in each source area (including area *A* itself) on average establishing the same number of synapses in the target area *A*. For each target area, the FLN in the network should add up to the total fraction of connections from vision-related cortical areas, which is not known a priori. For normalization, we consider also non-visual areas, for which distances are available and for which we can hence also estimate the FLN. The total fraction of all connections from subcortical regions averages 1.3% in eight cortical areas (Markov et al. 2011). This allows us to normalize the FLN from all cortical areas as $\sum _{B}\hbox {FLN}_{AB}=1-\hbox {FLN}_{\mathrm {i}}-0.013$, where the sum includes both modeled and non-modeled cortical areas. Combining the binary information on the existence of connections with the connection densities gives the area-level connectivity matrix with indegrees spanning five orders of magnitude (Fig. 4d).

**Fig. 5:**
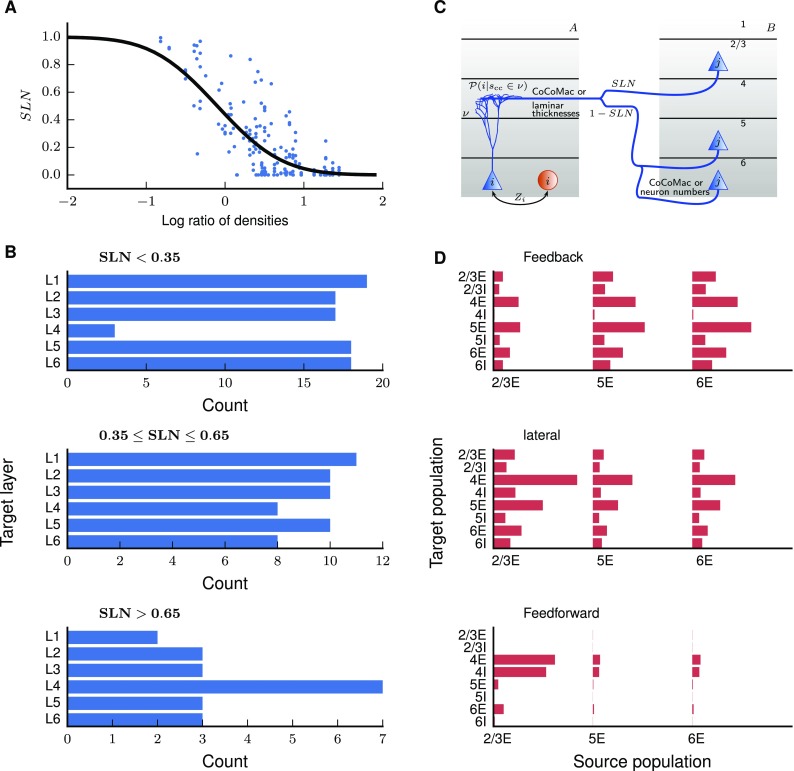
Layer- and population-specific cortico-cortical connection patterns. **a** Fraction of source neurons in supragranular layers (SLN) versus logarithmized ratio of the overall neuron densities of the two areas. SLN from Markov et al. (2014b), neuron densities from Hilgetag et al. (2016). Black curve, fit using a beta-binomial model (Eq. (1); $a_{0}=-\,0.152, \;a_{1}=-\,1.534, \;\phi =0.214$). **b** Laminar target patterns of synapse locations in relation to the SLN value of the source pattern. Target patterns are taken from the CoCoMac database (Felleman and Van Essen 1991; Barnes and Pandya 1992; Suzuki and Amaral 1994b; Morel and Bullier 1990; Perkel et al. 1986; Seltzer and Pandya 1994) and SLN data from Markov et al. (2014b) mapped to the FV91 scheme. **c** Illustration of the procedure (Supplementary Eq. 3) for distributing synapses across layers and populations. A source neuron from population *j* in area *B* sends an axon to layer *v* of area *A* where a cortico-cortical synapse $s_{\mathrm {CC}}$ is formed at the dendrite of a neuron from population *i*. The dendritic morphology is from Mainen and Sejnowski (1996) (source: http://NeuroMorpho.org; Ascoli et al. 2007). **d** Laminar patterns of cortico-cortical connections in the feedback, lateral, and feedforward direction, measured as the indegree of the population pairs divided by the sum of indegrees over all pairs, and then averaged across area pairs with the respective connection type ($K_{ij}=\langle K_{iA,jB}/\sum _{i^{\prime },j^{\prime }}K_{i^{\prime }A,j^{\prime }B}\rangle _{A,B}$). The categorization into feedback, lateral, and feedforward types follows the SLN value as in **b**

The distribution of cortico-cortical synapses across layers is based on layer-specific tracing results from CoCoMac and Markov et al. (2014b). We model cortico-cortical connections as purely excitatory, a good approximation to experimental findings (Salin and Bullier 1995; Tomioka and Rockland 2007). If available, CoCoMac data define the set of source layers; otherwise we include all layers except layer 4 in the source pattern. The synapses are distributed across the source layers according to the fractions of supragranular labeled neurons (SLN) from Markov et al. (2014b). Markov et al. (2014b) do not distinguish between the infragranular layers 5 and 6, so that between these source layers we either distribute synapses based on labeling density estimates from CoCoMac if available, or in proportion to the sizes of their excitatory populations. Since SLN data are not available for all connections, we supplement them with statistical estimates. To this end, we exploit a sigmoidal relation between the logarithmized ratios of cell densities of the participating areas and the SLN of their connection (as suggested by Beul et al. 2017). Following Markov et al. (2014b), we use a beta-binomial model for the fit, which employs a beta-binomial distribution of source neurons (Fig. 5a). The apparent deviation of the fit is caused by the high dispersion of the data. Surrogate data generated from the fitted distribution show the same apparent asymmetry around the sigmoidal curve as the experimental data, but for low dispersion, the surrogate data closely follow the fitted curve (Supplementary Fig. S1).

Combining target patterns from the CoCoMac database with source patterns from the data sets of Markov et al. (2014b), we find that synaptic target patterns depend on SLN (Fig. 5b). Figure 5b shows the accumulated layer-specific numbers of projections. The termination patterns vary substantially between individual connections. Overall, connections with high SLN preferentially form synapses in the granular layer 4 while low SLN is associated with termination patterns avoiding layer 4, and intermediate SLN with an approximately uniform distribution of synaptic locations across the six layers of cortex. This result refines the classification of Felleman and Van Essen (1991), in which all projection types can have a bilaminar origin, by showing that the termination pattern depends on the type of bilaminar origin (low, medium, or high SLN). We use this finding to derive target patterns where CoCoMac is incomplete. The systematic dependence of target on source patterns enables us to define classes of laminar projection patterns based on source patterns alone (cf. Markov et al. 2014b), instead of jointly considering source and target patterns as done in earlier work (Felleman and Van Essen 1991; Hilgetag et al. 2000). We denote projections with low, intermediate, and high SLN respectively as *feedback*, *lateral*, and *feedforward* projections. We take $\hbox {SLN}<0.35$ to correspond to feedback projections, $\hbox {SLN}>0.65$ to feedforward projections and $\hbox {SLN}\in [0.35,\;0.65]$ to lateral projections. The corresponding termination patterns $P_{\mathrm {t}}$ for connections without laminar information in CoCoMac are

$$\begin{aligned}&\{4\}\;\;\text {for }\;\;\hbox {SLN}>0.65\\&\{1,2/3,5,6\}\;\;\text {for }\;\;\hbox {SLN}<0.35\\&\{1,2/3,4,5,6\}\;\;\text {for }\;\;\hbox {SLN}\in [0.35,0.65], \end{aligned}$$

and we distribute synapses among the layers in the termination pattern in proportion to their thickness. Repetition of the analysis while varying the boundaries does not lead to qualitative differences (Supplementary Fig. S5). This confirms that the exact definition of the SLN boundaries between distinct laminar termination patterns does not critically influence the identified pathways.

The inclusion of layer 1 in the set of synaptic target layers is necessary for assigning synapses to neurons with cell bodies in the other layers, which enables characterizing polysynaptic paths. We statistically map synapse to cell body locations by taking into account the dendritic extent of the different cell types (Fig. 5c). For this, we compute the conditional probability $\mathcal {P}(i|s_{\mathrm {cc}}\in v)$ for the target neuron to belong to population *i* if a cortico-cortical synapse $s_{\mathrm {cc}}$ is in layer *v* (Supplementary Table S11), based on morphological reconstructions of cat V1 neurons (Binzegger et al. 2004). This probability equals the sum of probabilities that a synapse is established on the different Binzegger et al subpopulations making up our populations,

11 $$\begin{aligned} {\mathcal {P}}(i|s_{\rm {cc}}\in v)={\mathcal {P}}\left( \bigcup_{{\rm {c}}_{\rm {B}}\in i}c_{\rm {B}}|s_{\rm {cc}}\in v\right) =\sum _{c_{\rm {B}}\in i}{\mathcal {P}}(c_{\rm {B}}|s_{\rm {cc}}\in v), \end{aligned}$$

where

$$\begin{aligned} \mathcal {P}(c_{\mathrm {B}}|s_{\mathrm {cc}}\in v)=\frac{\mathcal {P}(c_{\mathrm {B}}\bigcap s_{\mathrm {cc}}\in v)}{\mathcal {P}(s_{\mathrm {cc}}\in v)}. \end{aligned}$$

The numerator gives the joint probability that a cortico-cortical synapse is formed in layer *v* on cell type $c_{\mathrm {B}}$,

12 $$\begin{aligned} \mathcal {P}(c_{\mathrm {B}}\bigcap s_{\mathrm {cc}}\in v)=\frac{N^{\mathrm {syn,CC}}(v,c_{\mathrm {B}})\mathcal {P}(c_{\mathrm {B}})}{\sum _{v^{\prime },c_{\mathrm {B}}^{\prime }}N^{\mathrm {syn,CC}}(v{}^{\prime },c_{\mathrm {B}}^{\prime })\mathcal {P}(c_{\mathrm {B}}^{\prime })}, \end{aligned}$$

and the denominator is the probability of a cortico-cortical synapse in layer *v*, computed by summing over cell types,

$$\begin{aligned} \mathcal {P}(s_{\mathrm {cc}}\in v)=\sum _{c_{\mathrm {B}}}\mathcal {P}(c_{\mathrm {B}}\bigcap s_{\mathrm {cc}}\in v). \end{aligned}$$

$N^{\mathrm {syn,CC}}(v,c_{\mathrm {B}})$ represents the number of cortico-cortical synapses in layer *v* on cell type $c_{\mathrm {B}}$ in the data set of Binzegger et al. (2004),

$$\begin{aligned} N^{\mathrm {syn,CC}}(v,c_{\mathrm {B}})=\mathcal {P}(s_{\mathrm {cc}}|c_{\mathrm {B}}\bigcap s\in v)\,N^{\mathrm {syn}}(v,c_{\mathrm {B}}). \end{aligned}$$

Note that this does not equal the (population-specific) number of cortico-cortical synapses in our model, but is only used to compute the probability of targeting a particular cell type in a particular layer according to Eq. (12). These equations lead to laminar connectivity patterns which differ from the synaptic laminar patterns (Fig. 5c). The resulting laminar distributions of target cell bodies are nevertheless distinct between feedforward, lateral, and feedback projections (Fig. 5d). While feedback projections establish synapses outside L4, they also reach L4 neurons that have apical dendrites in the supragranular layers. Assigning synapses according to the neuron morphologies even results in L4 excitatory neurons receiving more feedback than L2/3 neurons, since the total length of the apical dendrites of L4 pyramidal cells in L2/3 exceeds that of the dendrites of L2/3 neurons. Similarly, infragranular neurons receive a small amount of feedforward input via their apical dendrites in L4.

Furthermore, we take into account that in cortico-cortical feedback connections, a disproportionately high number of synapses is formed onto excitatory neurons (Johnson and Burkhalter 1996; Anderson et al. 2011). For each feedback connection in the model, we redistribute the synapses across the excitatory and inhibitory target populations and determine $Z_{i}$ such that 93% of synapses in each cortico-cortical projection are established on excitatory neurons.

Combining the considerations above, we obtain the number of cortico-cortical (type III) synapses from excitatory population *j* of area *B* to population *i* of area *A* (cf. Fig. 5c), as summarized in mathematical form in Supplementary Eq. 3.

This concludes the derivation of the network connectivity (Supplementary Fig. S3). We summarize the heuristics used to complete the experimental data along with starting points for more detailed derivations in Table 2. Averaged across all populations and areas, neurons receive 50.1% of their inputs from local neurons within the same $1\,\mathrm {mm}^{2}$ patch, 18.1% from cortico-cortical inputs, 28.5% from neurons local to the area but outside of the $1\,\mathrm {mm}^{2}$ patch, and 3.3% from neurons in non-visual cortical and non-cortical regions. The latter two contributions are treated as external inputs in the context of this study.

**Fig. 6:**
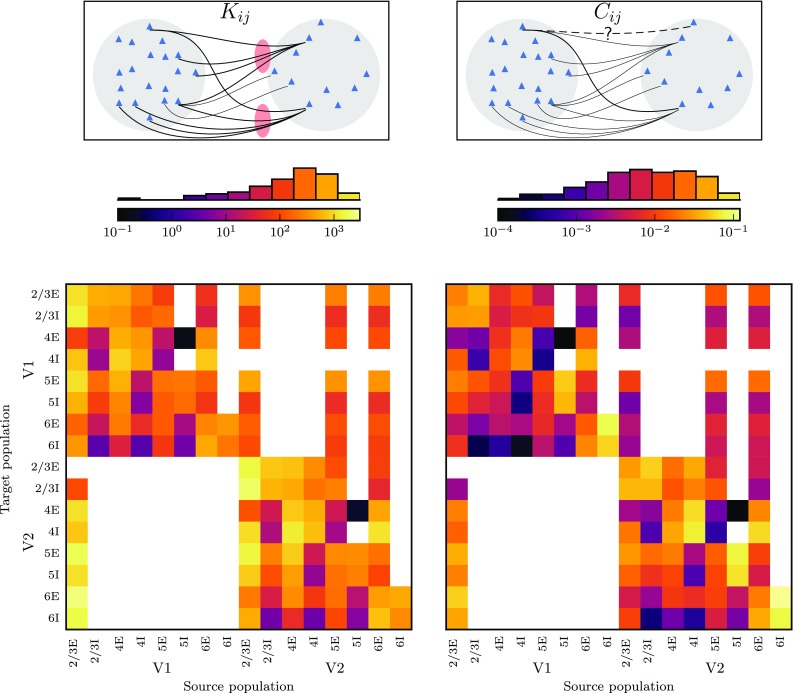
Population sizes matter for connectivity. Connectivity within and between areas V1 and V2 computed as pairwise indegrees (left) and connection probabilities (right). The latter are defined as the probability of $\ge 1$ synapse between any pair of source and target neurons, and can be obtained in linear approximation from the former by dividing by the size of the source population. The histograms show the occurrence of values in the bins defined by the color scales

Combining the numbers of synapses with the population sizes makes it possible to translate between different measures of connectivity. For instance, connectivity is often described in terms of connection probabilities (cf. Eq. 2). Other frequently used measures are the in- and outdegrees of the connections, respectively corresponding to the number of synapses that a neuron in the target (source) population receives (sends) (cf. Eqs. 3, 4). Time-averaged spiking rates, a first-order dynamical measure, depend to a large extent on the indegrees of the connections to the target population. On the other hand, for measuring and interpreting correlation, a second-order measure, the probability of the connection is the most relevant. Figure 6 shows a subset of the network connectivity expressed in terms of indegrees and in terms of connection probabilities. Note the differences between the measures, for instance when comparing the connections 2/3E$\rightarrow$4E and 2/3I$\rightarrow$4E in both areas. The indegrees of the two connections are substantially different, while the connection probability is very similar since it takes into account the fact that 4E contains more neurons than 4I. This dependence on population size also leads to differently shaped distributions of indegrees and connection probabilities, connection probabilities being more broadly distributed than indegrees.

### Area-level community structure relates to functional organization

**Fig. 7:**
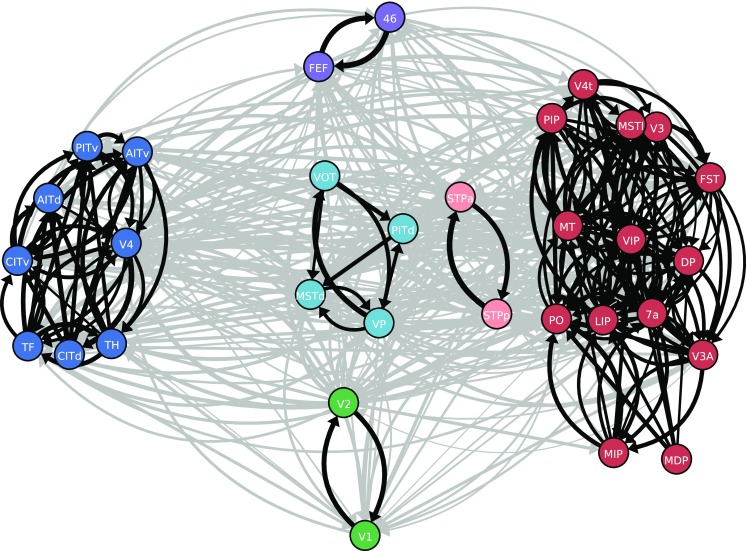
Community structure of the network. Clusters in the connectivity graph, indicated by the color of the nodes: lower visual areas (green), dorsal stream areas (red), superior temporal polysensory areas (light red), mixed cluster containing areas VP, VOT, PITd and MSTd (light blue), ventral stream (dark blue), and frontal areas (purple). Black, connections within clusters; gray, connections between clusters. Line thickness encodes logarithmized outdegrees. Only edges with relative outdegree $>10^{-3}$ are shown. For visual clarity, clusters are spatially segregated and inside clusters, areas are positioned using a force-directed algorithm (Kamada and Kawai 1989)

We test if the network follows known organizing principles by analyzing the community structure in the weighted and directed graph of area-level connectivity. The map equation method (Rosvall et al. 2009) applied on the outdegrees reveals six clusters (Fig. 7). We test the significance of the corresponding modularity $Q=0.38$ by comparing with 1000 surrogate networks conserving the total outdegree of each area by shuffling its targets. This yields $Q=-0.03\pm 0.03$, indicating the significance of the clustering. The anatomical community structure shows a correspondence with known functional groupings. Two large clusters comprise ventral stream areas along with parahippocampal areas TH and TF, and dorsal stream areas, respectively. The grouping of areas TF and TH with ventral stream areas is reasonable in view of the involvement of these parahippocampal areas in object and spatial memory processes (Bachevalier and Nemanic 2008; Nemanic et al. 2004) and was also obtained for the binary connection matrix of Felleman and Van Essen (1991) containing about half of the connections present in our weighted connectivity matrix (Hilgetag et al. 2000). The polysensory dorsal stream areas STPp and STPa have strong recurrent connections and are thus grouped outside of the large dorsal stream cluster. Ventral areas VOT and PITd are grouped with early visual area VP and dorsal area MSTd. Early visual areas V1 and V2 form a separate cluster, as do the two frontal areas FEF and 46. Nonetheless, the clusters are heavily interconnected (Fig. 7). The basic separation into ventral and dorsal clusters matches that found for the binary connection matrix of Felleman and Van Essen (1991) (Jouve et al. 1998; Hilgetag et al. 2000), but there are also important differences. For instance, our clustering groups area 7a with the dorsal instead of the ventral stream, better matching the scheme described by Nassi and Callaway (2009), and early visual areas V1 and V2 as well as frontal areas 46 and FEF are placed in separate clusters, respectively, in line with the differential functional properties of these areas and their non-unique association with the dorsal and ventral streams. The community structure is robust against excluding a small percentage ($\sim$10%) of the experimental data of Markov et al. (2014a) that underlie the fit of the exponential relation between connection densities and inter-areal distance and estimating the connection densities of these connections from the fit (Supplementary Fig. S4). Furthermore, the community structure is robust against adding a random fluctuation to the estimated FLN on the order of the spread of the experimental data around the fit in Fig. 4a (Supplementary Fig. S4).

### Path analysis of the connectivity graph

**Fig. 8:**
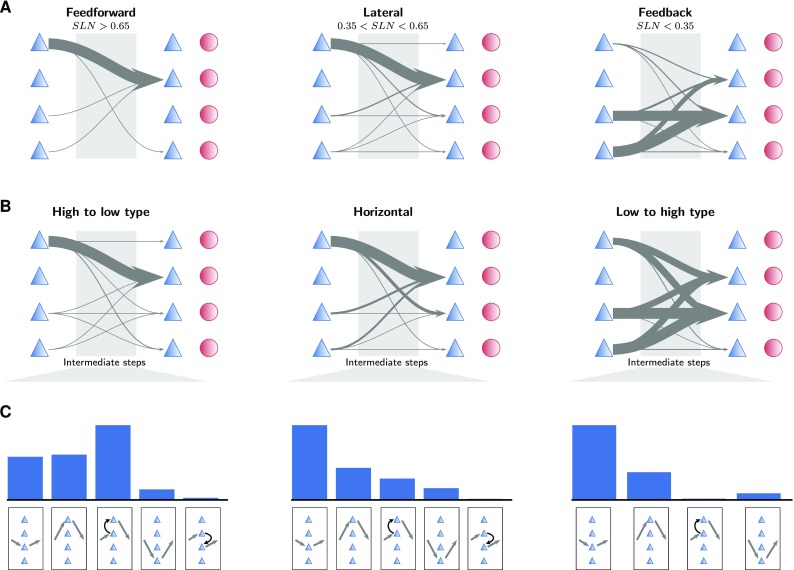
Population specificity organizes paths hierarchically and structurally. **a** Population-specific patterns of shortest paths between directly connected pairs of areas categorized according to their hierarchical relation as defined by fractions of supragranular labeled neurons (SLN). Arrow thickness indicates the relative occurrence of the particular pattern. The symbols mark excitatory (blue triangles) and inhibitory (red circles) populations stacked from L2/3 (top) to L6 (bottom). **b** Population-specific patterns of shortest paths between all pairs of areas categorized according to the difference between their architectural types. Arrow thickness indicates the occurrence of the particular pattern. **c** Occurrence of population patterns in areas that appear in the intermediate stage in the shortest path between two areas

To investigate the implications of the derived connectivity for the communication between areas, we detect the shortest paths between pairs of areas in the network (see “Materials and methods”). The shortest paths between cortical areas follow distinct patterns depending on the structural and hierarchical relation between the areas. The laminar patterns of the shortest paths between directly connected areas depend on the hierarchical relation between the areas (Fig. 8a). In the feedforward direction, shortest paths predominantly start in 2/3E and end in 4E, as expected from the layer-specific connectivity (Fig. 5). Lateral shortest paths similarly mostly originate in 2/3E and terminate in 4E. Feedback paths, on the other hand, mostly start in the infragranular layers 5 and 6 and target neurons in layers 4 and 5.

Taking all pairs of areas into account regardless of whether they are directly connected, a similar picture emerges (Fig. 8b). Since SLN are only available for directly connected areas, we here group pairs of areas according to the difference between their architectural types. We call pathways from structurally differentiated to less differentiated areas ‘high-to-low-type’, those in the opposite direction ‘low-to-high-type’ and those between structurally similar areas ‘horizontal’. High-to-low-type pathways as well as horizontal pathways follow the 23E$\rightarrow$4E pattern. Low-to-high-type pathways, on the other hand, are more uniformly distributed with most paths starting in 5E or 6E, and ending in 4E or 5E. These observations consider only the first and last populations of the entire path. However, 45% of the shortest paths take a detour via one or multiple intermediate areas. Even if the two areas are directly connected, the direct connection is not the shortest (strongest) path in 10% of the cases. In intermediate areas, the shortest paths involve one or two populations. From high-type to low-type areas, these intra-area paths are mostly from 4E to 2/3E (Fig. 8c), in line with the start-end pattern shown in Fig. 8b, but a substantial fraction passes through 2/3E and 5E only. Indirect, horizontal paths mostly involve a relay via 5E, and to a lesser extent 2/3E and the 4E$\rightarrow$2/3E pattern. Similarly, connections from low-type to high-type areas are mostly forwarded by the 5E population only. These results suggest that directionally distinct paths in the population-level connectivity open up communication channels for specifically targeted cortico-cortical communication across sets of areas. We test the robustness of these findings against altering the SLN thresholds used for the hierarchical categorization of connections and against pruning of the SLN data underlying the network construction, including the sigmoidal fit (Fig. 5a). We found no qualitative variations in the paths between areas, meaning that our findings are independent of moderate variations in the underlying data and heuristics (Supplementary Fig. S5 and Supplementary Fig. S6). Furthermore, the laminar patterns of shortest paths remain qualitatively unchanged when only connections with available experimental SLN data are included in the analysis (Supplementary Fig. S7).

## Discussion

The present study integrates data on cortical architecture, geometry, and connectivity into a comprehensive unihemispheric network description of the vision-related areas of macaque cortex. A number of simplifying assumptions and heuristics that are based on established and novel statistical regularities complement the measurements in view of the sparseness of quantitative species- and area-specific data. Our study thus represents a compromise between detail and conciseness, where avenues for future improvements are explicitly identified. The multi-scale network description consists of a population-, layer- and area-specific connectivity map together with neural population sizes, which resolve ambiguities in connectivity measures. In the derived connectivity, we find multiple clusters reflecting the anatomical and functional partition of visual cortex into early visual areas, ventral and dorsal streams, and frontal areas, showing that the network construction yields a meaningful structure. The laminar resolution of the model, along with a statistical mapping of synapse to target cell body locations, enables a novel characterization of direct and indirect paths across neural populations in the cortex. Our findings stand up to validation with varied network models defined based on moderately pruned connectivity data and models where the employed heuristics are relaxed.

The cortico-cortical connectivity is based on axonal tracing data collected in a new release of CoCoMac (Bakker et al. 2012) combined with recent quantitative and layer-specific retrograde tracing experiments (Markov et al. 2014b, a). The projections revealed by these axonal tracing data are complex and not strictly sequential, including bypass connections such as those from V1 to V4 bypassing V2 (Nakamura et al. 1993). To translate FLN data into connection densities, we assume that the number of synapses established in the target area does not differ across projecting areas. Implicitly, other studies that interpret FLN in terms of connection strengths (e.g., Markov et al. 2013; Goulas et al. 2014) make the same assumption. There is, however, evidence that the number of cortico-cortical synapses per neuron in a projection depends on its direction (Rockland 2003).

We fill in missing data using relationships between laminar source and target patterns (Felleman and Van Essen 1991; Markov et al. 2014b) and architectural differentiation (Hilgetag et al. 2016; Beul et al. 2017), an approach for which Barbas (1986) and Barbas and Rempel-Clower (1997) laid the groundwork. To estimate missing data on connection densities, we use the exponential decay of FLN with inter-areal distance, which relies on the exponential distribution of axon lengths combined with the parcellation of cortical space into areas (Ercsey-Ravasz et al. 2013). For simplicity, we here assume an isotropic distribution of connection densities, in line with Ercsey-Ravasz et al. (2013), but data from hamster cortex suggest that axons may extend further along the mediolateral axis than along the anterior–posterior axis (Cahalane et al. 2011).

The use of axonal tracing results avoids the pitfalls of tractography based on diffusion MRI data, which strongly depends on parameter choices (Thomas et al. 2014), has limited spatial resolution, cannot sense the direction of connections, and has been found to both underestimate (Calabrese et al. 2015b) and overestimate (Maier-Hein et al. 2016) cortical connectivity. A recent study comparing dMRI-based tractography on macaque cortex with retrograde tracing data shows that tractography after removal of false positives and false negatives is modestly informative about connection strengths (Donahue et al. 2016). Since axonal tracing data need to be combined across individuals whereas dMRI maps are obtained in individual brains, the two approaches are complementary.

The local connectivity of our network customizes that of the microcircuit model of Potjans and Diesmann (2014) according to the specific architecture of each area, taking into account neuronal densities and laminar thicknesses. Although the model of Potjans and Diesmann (2014) is based on data from rat and cat cortex, it serves as a prototype for the local circuits in our study due to the lack of similarly comprehensive quantitative data on pairwise connection probabilities in macaque cortex. Future revisions of the model can refine the analysis by incorporating additional knowledge on the local structure of macaque cortex as it becomes available, for instance information on cell morphologies in different areas (e.g.,  Gilman et al. 2017). Neuronal densities decrease from high to low-type visual areas, resulting in an apparent caudal-to-rostral gradient (Charvet et al. 2015). Combined with the assumption of a constant volume density of synapses (O’Kusky and Colonnier 1982; Cragg 1967) this yields higher indegrees in low-type areas. This trend matches an increase in dendritic spines per pyramidal neuron (Elston and Rosa 2000; Elston 2000; Elston et al. 2011). We thus clarify how volume densities of neurons and synapses together determine such an increase in per-neuron connectivity along the architectonic gradient of visual areas.

Total cortical thickness decreases with overall neuron density (cf., von Economo and Van Bogaert 1927; la Fougère et al. 2011; Cahalane et al. 2012). Similarly, total thicknesses from MR measurements decrease with increasing architectural type (Wagstyl et al. 2015), which has a strong positive correlation with cell density (Hilgetag et al. 2016). Laminar and total cortical thicknesses are determined from micrographs, which has the drawback that this approach covers only a small fraction of the surface of each cortical area. For absolute, but not relative, thicknesses, another caveat is potential shrinkage and obliqueness of sections. It has also been found that laminar and total thicknesses depend on the sulcal or gyral location of areas, which is not offset by a change in neuron densities (Hilgetag and Barbas 2006). However, regressing our relative thickness data against cortical depth of the areas registered to F99 revealed no significant trends of this type (Supplementary Fig. S2). Laminar thickness data are surprisingly incomplete, considering that this is a basic anatomical feature of cortex. Total thicknesses have already recently been measured across cortex (Calabrese et al. 2015a; Wagstyl et al. 2015), and could complement the data set used here covering 14 of the 32 areas. However, when computing numbers of neurons, using histological data may be preferable, because shrinkage effects on neuronal densities and laminar thicknesses partially cancel out.

We statistically assign cortico-cortical synapses to target neurons based on anatomical reconstructions (Binzegger et al. 2004). This assumes that the anatomical strength of a connection between two different types of neurons depends on the product of the average number of synapses formed by the source neuron in a particular layer and the dendritic density of the target neurons in that layer, an extended version of Peters’s rule (Braitenberg and Schüz 1991). Axo-dendritic overlap predicts connectivity to some extent, but the actual multiplicity and synaptic strength of connections between individual neurons show large variations (Shepherd et al. 2005; Kasthuri et al. 2015). However, Rees et al. (2016) review existing literature and conclude that using Peters’s rule at the level of cell types instead of individual cells can deliver a reasonable approximation to cortical circuitry. On the target side, the assignment of synapses based on dendritic extent yields laminar cell body distributions for feedforward and feedback projections that mostly follow the classical scheme for laminar synapse distributions of Felleman and Van Essen (1991). However, in our network, layer 4 neurons receive substantial feedback input, stressing the importance of distinguishing between synapse and cell body positions, as previously pointed out by De Pasquale and Sherman (2011). This prediction can be tested for example with glutamate uncaging in the source area combined with patch-clamp recording in the target area (Covic and Sherman 2011), or via axonal tracing combined with morphological reconstruction of the target neurons (Porter 1997). Covic and Sherman (2011) found feedback onto layer 4 neurons in mouse auditory cortex; however, such a pattern remains to be shown in primates. This finding would shed a new perspective on the role of L4 neurons in cortical processing. In predictive coding for instance, L4 neurons are hypothesized to process forward prediction errors using their feedforward inputs, while layer 5 pyramidal cells process feedback predictions via their apical dendrites in the supragranular layers (Bastos et al. 2012). With L4 neurons receiving additional feedback via dendrites reaching into layer 2/3, their role could be more intricate and involve processing of both feedforward and feedback signals.

Our analysis includes target patterns from the CoCoMac database, which enables us to link target patterns to quantitatively defined laminar projection patterns of bilaminar origin, refining the classification of Felleman and Van Essen (1991). Markov et al. (2014b) combined their source patterns from retrograde tracing with target patterns from previous anterograde tracing studies in different species and distinguished feedback and feedforward connections further into hierarchically short-range and long-range projections, respectively. They found subtle differences in target patterns, e.g., that feedforward connections from high-type visual areas terminate in layers 3B and 4 of intermediate areas, but exclusively in layer 4 in low-type areas. However, the anterograde data used by Markov et al. (2014b) cover target patterns for connections in only a small subset of visual areas. Our data from CoCoMac include target patterns for all visual areas with 29% coverage of all connections in our network, but do not allow us to draw conclusions on such a fine classification into hierarchically short-range and long-range connections. Future work could test if a revised version of the full CoCoMac dataset using a finer layer distinction supports the findings of Markov et al. (2014b). Laminar specificity of cortico-cortical connections is important because it can support complementary channels for feedforward and feedback communication in cortex (Bastos et al. 2015b). In particular, anatomical segregation of communication channels likely plays a role in enabling directional differences in oscillation frequencies associated with inter-area communication (van Kerkoerle et al. 2014; Bastos et al. 2015a; Michalareas et al. 2016). This segregation can occur even in single cells that combine feedback and feedforward processing on their apical and basal dendrites (Körding and König 2001; Urbanczik and Senn 2014), again stressing the importance of taking cell morphologies into account.

The connectivity of neuronal networks can be described in terms of different measures, each highlighting a specific aspect of the network and relating differently to its dynamics. For instance, in mean-field descriptions of network dynamics, indegrees tend to be most directly related to stationary firing rates, while fluctuations around this stationary state depend on the population size, and therefore, correlations are determined by a combination of indegrees and connection probabilities (Brunel 2000; Helias et al. 2013). On the other hand, outdegrees relate more directly to the overall influence of each node. Our network description consisting of population sizes and numbers of synapses for each connection allows us to translate between these measures, showing how they differ in their relative strength across connections. Using the appropriate connectivity measures can facilitate the interpretation of observed dynamics.

The population-level connectivity enables us to identify the most prominent laminar projection patterns in shortest paths between areas. While pathways from high-type to low-type areas and horizontal pathways (between structurally similar areas) both follow a stereotypical pattern originating in the supragranular layers and targeting layer 4, projections from low-type to high-type areas feature a richer repertoire of layer-specific paths. At relay stages in indirect paths, horizontal pathways more closely resemble low-to-high-type pathways. These findings suggest that areas of equal architectural type communicate via similar pathways as connections from structurally more differentiated to less differentiated areas in terms of their start-end pattern, but that these pathways are often relayed via pathways similar to those from structurally less differentiated to more differentiated areas. The hypothesis that dynamical interactions follow these anatomical paths could be tested in experiments as well as numerical simulations. The anatomical paths in our model are fairly independent of whether they are categorized based on SLN or the architectural types. An exception is that a significant number of low-to-high-type paths originate in supragranular layers, while the origin of feedback paths is strongly dominated by the infragranular layers. Still, these similarities suggest that functional signatures of connections categorized based on the structural gradient are similar to those observed for hierarchical projections (van Kerkoerle et al. 2014; Bastos et al. 2015a; Michalareas et al. 2016).

We here concentrate on aspects of cortical structure for which substantial datasets are available, leaving aside insights on specific details in individual areas for which the available information is highly incomplete. Our algorithmic approach makes the network amenable to the integration of additional details, such as more diverse neuronal populations (Defelipe et al. 1999; Binzegger et al. 2004; Markram et al. 2015), additional area specificity of local circuits (Beul and Hilgetag 2015), connectivity patterns beyond pairwise connection probabilities (Song et al. 2005; Kasthuri et al. 2015; Markram et al. 2015), or spatial properties of connectivity (Colby et al. 1988; Salin et al. 1989; Gattass et al. 1997; Markov et al. 2014b). The cortico-cortical connectivity may be further refined by incorporating a dual counterstream organization of feedforward and feedback connections (Markov et al. 2014b), by including different numbers of cortico-cortical synapses per neuron in feedforward and feedback directions (Rockland 2003), and by incorporating cortico-cortical projection patterns on the single-cell level as found in mouse V1 (Han et al. 2017). It is also worth investigating whether the preferential targeting of excitatory neurons by feedback projections is part of a more gradual reduction in inhibition–excitation ratio from feedforward to feedback projections, as is the case for optogenetically determined EPSCs (D’Souza et al. 2016).

In this study, we concentrate on the network of vision-related areas within one hemisphere of cortex, thereby leaving aside callosal and subcortical connections as well as connections with other cortical areas. Since most tracing studies concentrate on one hemisphere, knowledge about callosal connections is sparse; however, tracing data from mouse cortex (Goulas et al. 2017) and rhesus monkey prefrontal cortex (Barbas et al. 2005) suggest similar construction principles to those of ipsilateral connections, which can be used to inform a future revision of the model. The integration of thalamo-cortical loops is an important extension of the model, but in view of the added complexity beyond the scope of the current study. Since the corresponding connectivity has been measured for parts of cortex only, it would be necessary to fill gaps in the data by empirical regularities similar to those used in the present study, possibly employing more advanced graph-theoretical techniques similar to Jouve et al. (1998). This would help ensure the realism of graph-theoretical properties of the connectivity matrix not tested for in the present study, and would enhance the reliability of individual entries of the matrix that are currently only first-order estimates.

Our study can thus be the starting point for iterative refinement and more detailed descriptions of cortical connectivity, contributing to a better understanding of cortical structure. It also provides the basis for numerical simulations that investigate the relation between structure and dynamics (Schmidt et al. 2016; Schuecker et al. 2017). In contrast to previous simulation studies, which are based on binary or coarsely weighted tracing data or on diffusion MRI (Honey et al. 2007; Knock et al. 2009; Deco et al. 2009), the weighted and directed graph resulting from our integration of axonal tracing data enables studying the activity supported by the highly heterogeneous connectivity of cortex.

## Electronic supplementary material

Below is the link to the electronic supplementary material.

Supplementary material 1 (pdf 1745 KB)
