# Supplementary material for: Multi-scale account of the network structure of macaque visual cortex
Source: Brain Struct Funct. 2017 Nov 16;223(3):1409–35. doi: 10.1007/s00429-017-1554-4 (PMC5869897; doi:10.1007/s00429-017-1554-4)
Supplement: Supplementary file 1 — Supplementary material 1 (pdf 1745 KB) [file 429_2017_1554_MOESM1_ESM.pdf]

Noname manuscript No.
(will be inserted by the editor)

Supplemental material to "Multi-scale account of the network structure of macaque visual cortex"

Maximilian Schmidt · Rembrandt Bakker · Claus C. Hilgetag · Markus Diesmann · Sacha J. van Albada

the date of receipt and acceptance should be inserted later

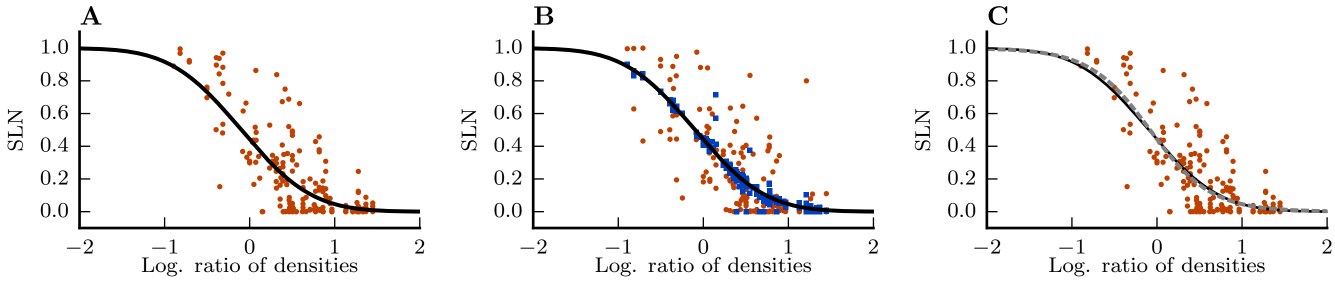

Figure S1: Related to Fig. 5. **Control for fit of a beta-binomal function**. Black curve in all panels, fit using a beta-binomial model with probit sigmoidal function (eq (7) in manuscript) ($a_0 = -0.152$, $a_1 = -1.534$, $\phi = 0.214$). (A): Red dots, real data from Markov et al (2014b). (B): Red dots, random data generated from a beta-binomial distribution with fitted parameters $a_0 = -0.152$, $a_1 = -1.534$, $\phi = 0.214$. Blue boxes, random data generated from a beta-binomial distribution with parameters $a_0 = -0.152$, $a_1 = -1.534$, $\phi = 0.001$. (C): Red dots, real data from Markov et al (2014b). Gray dashed curve, fit using a beta-binomial model with logit sigmoidal function.

M. Schmidt
Institute of Neuroscience and Medicine (INM-6) and Institute for Advanced Simulation (IAS-6) and JARA Institute Brain Structure-Function Relationships (JBI-1 /INM-10), Jülich Research Centre, Jülich, Germany
phone: +49-2461-61-4748. fax: +49-2461-61-9460
E-mail: maximilian.schmidt@riken.jp

R. Bakker
Institute of Neuroscience and Medicine (INM-6) and Institute for Advanced Simulation (IAS-6) and JARA Institute Brain Structure-Function Relationships (JBI-1 /INM-10), Jülich Research Centre, Jülich, Germany,
Donders Institute for Brain, Cognition and Behavior, Radboud University Nijmegen, Netherlands

C.-C. Hilgetag
Department of Computational Neuroscience, University Medical Center Eppendorf, Hamburg, Germany,
Department of Health Sciences, Boston University, USA

M. Diesmann
Institute of Neuroscience and Medicine (INM-6) and Institute for Advanced Simulation (IAS-6) and JARA Institute Brain Structure-Function Relationships (JBI-1 /INM-10), Jülich Research Centre, Jülich, Germany
Department of Psychiatry, Psychotherapy and Psychosomatics, Medical Faculty, RWTH Aachen University, Aachen, Germany,
Department of Physics, Faculty 1, RWTH Aachen University, Aachen, Germany

S. J. van Albada
Institute of Neuroscience and Medicine (INM-6) and Institute for Advanced Simulation (IAS-6) and JARA Institute Brain Structure-Function Relationships (JBI-1 /INM-10), Jülich Research Centre, Jülich, Germany

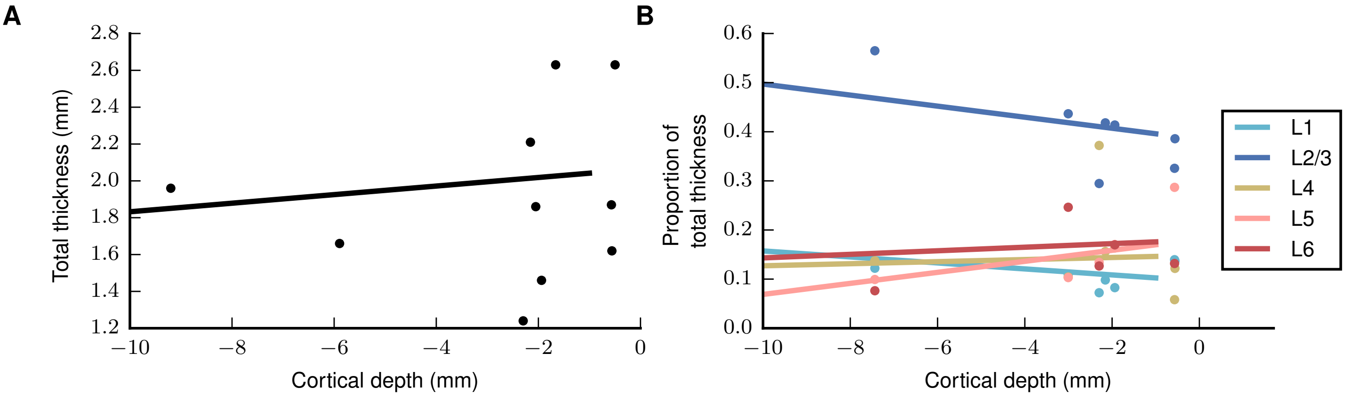

Figure S2: Related to Fig. 2. **Thickness versus cortical depth.** (A) Total thickness vs. cortical depth and linear least-squares fit showing no significant correlation ($r = 0.12$, $p = 0.68$). (B) Relative laminar thicknesses vs. cortical depth and linear least-squares fits also showing no significant correlation (L1: $r = -0.43$, $p = 0.14$, L2/3: $r = -0.46$, $p = 0.11$, L4: $r = 0.08$, $p = 0.79$; L5: $r = -0.53$, $p = 0.09$, L6: $r = 0.14$, $p = 0.69$). The thickness data is the same as in Fig. 4. Cortical depth data obtained from F99 surface statistics available through the Caret Software (Van Essen, 2012). Values for each area are averaged across cortical surface and both hemispheres. The data is obtained using the F99 Sulcal depth tool on http://cocomac.g-node.org and can be directly accessed via these two links: `http://cocomac.g-node.org/cocomac2/services/f99_sulcal_depth.php?atlas=FV91&shape=Depth-Right&mode=avg&output=tsv&run=1` and `http://cocomac.g-node.org/cocomac2/services/f99_sulcal_depth.php?atlas=FV91&shape=Depth-Left&mode=avg&output=tsv&run=1`.

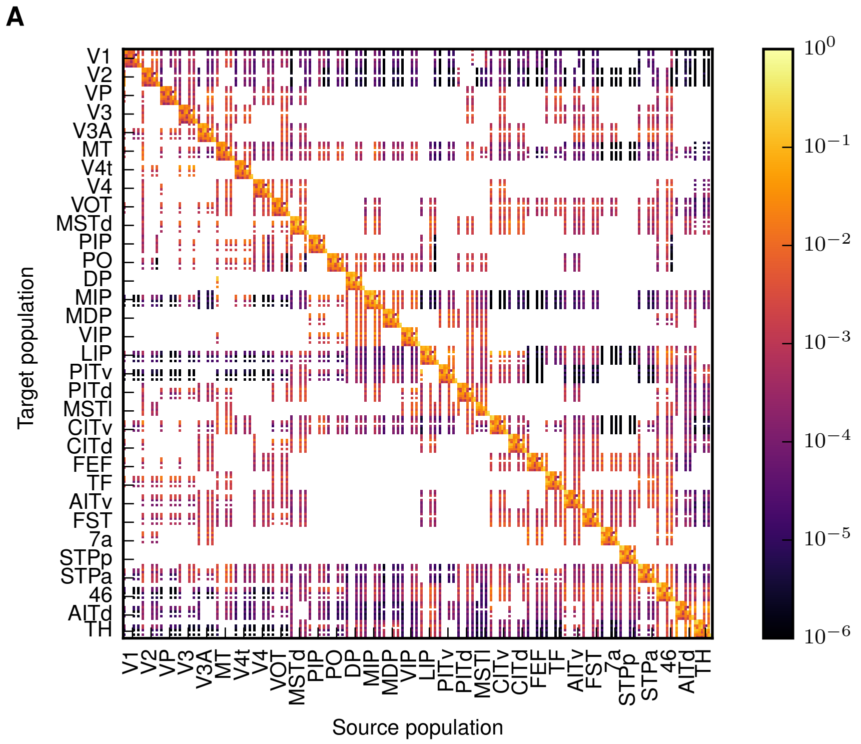

Figure S3: Related to Fig. 4. **Connection probabilities of the model** encoded in color. Areas are ordered according to their architectural types, and populations inside the areas are ordered as [2/3E, 2/3I, 4E/, 4I, 5E, 5I, 6E, 6I].

Cortical areas in the model

Lobe	Abbreviation	Brain Region
Occipital	V1	Visual area 1
	V2	Visual area 2
	V3	Visual area 3
	VP	Ventral posterior
	V3A	Visual area V3A
	V4	Visual area 4
	VOT	Ventral occipitotemporal
	V4t	V4 transitional
	MT	Middle temporal
Temporal	FST	Floor of superior temporal
	PITd	Posterior inferotemporal (dorsal)
	PITv	Posterior inferotemporal (ventral)
	CITd	Central inferotemporal (dorsal)
	CITv	Central inferotemporal (ventral)
	AITd	Anterior inferotemporal (dorsal)
	AITv	Anterior inferotemporal (ventral)
	STPp	Superior temporal polysensory (posterior)
	STPa	Superior temporal polysensory (anterior)
	TF	Parahippocampal area TF
	TH	Parahippocampal area TH
Parietal	MSTd	Medial superior temporal (dorsal)
	MSTl	Medial superior temporal (lateral)
	PO	Parieto-occipital
	PIP	Posterior intraparietal
	LIP	Lateral intraparietal
	VIP	Ventral intraparietal
	MIP	Medial intraparietal
	MDP	Medial dorsal parietal
	DP	Dorsal prelunate
	7a	7a
Frontal	FEF	Frontal eye field
	46	Middle frontal area 46

Table S1: List of areas in the model. All vision-related areas of macaque cortex in the parcellation of Felleman and Van Essen (1991).

Neuron densities

Layer-resolved neuronal volume densities for 14 areas are provided by H. Barbas (personal communication). For 3 of these areas, NeuN staining was used, and we linearly scale up the corresponding values to correct for undersampling with respect to Nissl staining as determined by repeat measurements of 11 areas. The original neuron densities resulting from NeuN staining are given in Hilgetag et al (2016a) Table 4.

Translation of Table 4 of Hilgetag et al (2016a)

**Area in** Hilgetag et al (2016a)	**FV91 area**	**Area in** Hilgetag et al (2016a)	**FV91 area**
V1	V1	MST	MSTd, MSTl
V2	V2	PIP	PIP
V3	V3	PIT	PITd, PITv
VP	VP	PO	PO
MT	MT	TF	TF
V3A	V3A	VIP	VIP
V4	V4	A46v	46
V4t	V4t	A7a	7a
VOT	VOT	AIT	AITd, AITv
CIT	CITd, CITv	FST	FST
DP	DP	STP	STPa, STPp
FEF	FEF	TH	TH
LIPd, LIPv	LIP	TEO*	PITd, PITv, VOT
TEr*	AITd, AITv, CITd, CITv		

Table S2: Scheme for translating architectural types, overall neuron densities and cortical thicknesses given in Table 4 of Hilgetag et al (2016a) to the modeled areas in the parcellation scheme of Felleman and Van Essen (1991). Entries marked with a star are used to translate the overall neuron density and cortical thickness which are not available in the finer of the two parcellations used by Hilgetag et al (2016a).

Relative laminar thicknesses from experimental literature

Area	1	2/3	4	5	6	Source
V1	0.08	0.25	0.37	0.14	0.16	O'Kusky and Colonnier (1982)
V1	0.09	0.29	0.39	0.11	0.12	Rakic et al (1991)
V1	0.08	0.32	0.38	0.14	0.08	Felleman et al (1997)
V1	0.05	0.31	0.36	0.14	0.14	Eggan and Lewis (2007)
V2	0.07	0.41	0.14	0.21	0.18	Markov et al (2014a)
V2	0.1	0.42	0.19	0.13	0.16	Rakic et al (1991)
V3	0.09	0.58	0.12	0.1	0.12	Markov et al (2014a)
V3	0.2	0.29	0.27	-	-	Angelucci et al (2002)
MT	0.11	0.54	0.13	0.11	0.11	Markov et al (2014a)
MT	0.09	0.43	0.14	0.16	0.18	Preuss and Goldman-Rakic (1991)
V4	0.09	0.53	0.12	0.12	0.12	Rockland (1992)
MIP	0.09	0.41	0.08	0.08	0.34	Rozzi et al (2006)
VIP	0.12	0.56	0.14	0.1	0.08	Preuss and Goldman-Rakic (1991)
LIP	0.09	0.36	0.09	0.08	0.39	Rozzi et al (2006)
LIP	0.13	0.52	0.12	0.13	0.1	Preuss and Goldman-Rakic (1991)
FEF	0.1	0.42	0.16	0.17	0.16	Boussaoud et al (1990)
TF	0.14	0.39	0.12	-	-	Preuss and Goldman-Rakic (1991)
FST	0.24	0.42	0.08	-	-	Lavenex et al (2002)
46	0.1	0.45	0.1	0.15	0.2	Eggan and Lewis (2007)
46	0.13	0.43	0.09	-	-	Petrides and Pandya (1999)
TH	-	-	0.0	-	-	Suzuki and Amaral (2003)
TH	0.14	0.33	0.12	0.29	0.13	Preuss and Goldman-Rakic (1991)

Table S3: Relative laminar thicknesses determined from the anatomical studies given in the last column.

Mapping of injection sites to FV91 parcellation

Monkey	M132 area	FV91 area	Monkey	M132 area	FV91 area
M88RH	V1	V1	M101LH	V2	V2
M121LH	V1	V1	M101RH	V2	V2
M81LH	V1	V1	M103LH	V2	V2
M85LH	V1	V1	M123LH	V4	V4
M85RH	V1	V1	M121RH	V4	V4
BB289RH	STPr	STPa	M119LH	TEO	V4
BB289LH	STPi	STPp	BB135LH	7A	7a
M90RH	STPc	STPp	M89LH	DP	DP
M106LH	9/46d	FEF	BB272RH	8l	FEF
M133LH	MT	MSTd	M116LH	46d	46
M116RH	9/46v	46	BB272LH	8m	FEF
M128RH	TEPd	CITv	M108LH	PBr	STPp

Table S4: Injected areas of the data set of Markov et al (2014a) in the M132 parcellation and corresponding areas in the FV91 scheme. Only the injections in vision-related cortex are shown.

Inter-areal distances

Area	V1	V2	VP	V3	V3A	MT	V4t	V4	VOT	MSTd	PIP	PO	DP	MIP	MDP	VIP
**V1**	0.0	17.9	19.9	14.6	16.8	22.5	23.1	22.9	29.0	26.8	18.8	21.5	23.7	24.5	29.2	26.3
**V2**	17.9	0.0	16.1	17.8	18.2	20.0	20.5	21.2	24.5	24.4	19.8	23.8	24.6	26.0	30.8	25.8
**VP**	19.9	16.1	0.0	20.8	19.0	14.9	15.1	14.6	12.8	20.9	20.1	25.2	25.4	26.9	31.9	25.5
**V3**	14.6	17.8	20.8	0.0	8.1	15.9	17.0	18.5	26.9	19.4	10.6	14.6	15.1	16.9	22.0	18.1
**V3A**	16.8	18.2	19.0	8.1	0.0	12.4	13.4	15.6	23.4	15.4	9.2	15.0	9.4	16.3	21.2	13.8
**MT**	22.5	20.0	14.9	15.9	12.4	0.0	6.0	11.4	13.7	10.0	13.2	19.1	16.6	20.0	23.9	13.7
**V4t**	23.1	20.5	15.1	17.0	13.4	6.0	0.0	9.9	12.1	12.1	15.2	21.3	17.8	22.1	26.3	16.4
**V4**	22.9	21.2	14.6	18.5	15.6	11.4	9.9	0.0	13.1	17.8	18.6	24.6	20.4	25.6	30.5	21.4
**VOT**	29.0	24.5	12.8	26.9	23.4	13.7	12.1	13.1	0.0	19.7	24.6	30.0	28.5	31.0	36.0	26.6
**MSTd**	26.8	24.4	20.9	19.4	15.4	10.0	12.1	17.8	19.7	0.0	14.5	20.6	17.1	20.2	24.1	11.5
**PIP**	18.8	19.8	20.1	10.6	9.2	13.2	15.2	18.6	24.6	14.5	0.0	9.5	12.3	9.8	14.2	11.2
**PO**	21.5	23.8	25.2	14.6	15.0	19.1	21.3	24.6	30.0	20.6	9.5	0.0	18.9	6.9	10.0	16.0
**DP**	23.7	24.6	25.4	15.1	9.4	16.6	17.8	20.4	28.5	17.1	12.3	18.9	0.0	18.3	22.0	11.1
**MIP**	24.5	26.0	26.9	16.9	16.3	20.0	22.1	25.6	31.0	20.2	9.8	6.9	18.3	0.0	6.3	14.6
**MDP**	29.2	30.8	31.9	22.0	21.2	23.9	26.3	30.5	36.0	24.1	14.2	10.0	22.0	6.3	0.0	17.4
**VIP**	26.3	25.8	25.5	18.1	13.8	13.7	16.4	21.4	26.6	11.5	11.2	16.0	11.1	14.6	17.4	0.0
**LIP**	27.8	27.6	28.0	19.3	14.6	16.0	18.6	23.3	29.1	12.4	13.6	19.5	10.1	18.2	21.1	7.4
**PITv**	32.9	28.0	16.8	30.4	26.8	16.3	14.8	16.3	7.4	21.5	27.6	32.9	31.9	34.0	38.7	28.8
**PITd**	31.6	27.3	17.8	27.4	23.5	13.1	11.6	14.1	8.6	18.7	24.8	30.6	28.2	31.4	35.9	25.7
**MSTl**	28.4	24.4	17.4	22.6	18.9	8.2	9.9	15.7	13.2	9.1	18.0	24.0	22.7	24.2	28.3	16.9
**CITv**	38.8	33.4	22.4	36.0	32.7	21.6	21.1	22.5	12.9	25.7	32.4	37.6	37.7	38.8	43.1	33.1
**CITd**	37.7	32.5	22.0	34.3	31.0	19.5	19.2	20.9	12.2	23.2	30.5	35.9	35.7	37.0	41.0	30.7
**FEF**	57.1	53.9	48.3	50.0	45.8	37.9	40.3	45.8	42.1	36.4	42.9	47.9	46.2	45.4	46.7	36.8
**TF**	29.6	24.8	16.3	27.4	24.7	15.8	17.1	20.1	14.4	20.2	23.5	28.0	29.8	29.5	33.7	25.9
**AITv**	43.8	38.2	28.2	41.1	38.1	26.6	27.0	28.9	19.5	30.1	36.7	41.4	42.7	42.5	46.7	36.5
**FST**	33.7	28.9	20.3	28.8	25.6	14.2	15.7	19.2	12.8	16.5	24.6	29.8	29.6	30.6	34.2	23.8
**7a**	28.2	27.6	27.4	19.9	14.5	15.5	18.0	22.4	27.8	11.0	14.5	20.9	11.5	20.0	23.2	9.4
**STPp**	38.0	34.3	27.7	31.7	28.1	18.1	20.0	25.5	22.5	16.0	27.2	32.9	30.8	33.0	36.6	24.3
**STPa**	44.3	39.2	30.2	40.2	37.1	25.5	27.0	30.0	21.8	27.3	35.3	40.4	40.9	41.0	44.5	33.6
**46**	62.9	59.5	54.1	55.9	51.8	43.9	46.4	51.5	47.7	42.4	49.0	53.3	52.0	50.8	52.0	42.6
**AITd**	46.3	40.9	31.2	43.3	40.4	28.4	29.0	30.3	21.6	31.6	39.0	43.9	44.7	44.7	48.9	38.1
**TH**	30.8	26.3	19.9	27.5	25.1	17.1	18.9	22.6	18.1	20.6	22.4	26.2	29.4	28.2	31.4	24.9

Area	LIP	PITv	PITd	MSTl	CITv	CITd	FEF	TF	AITv	FST	7a	STPp	STPa	46	AITd	TH
**V1**	27.8	32.9	31.6	28.4	38.8	37.7	57.1	29.6	43.8	33.7	28.2	38.0	44.3	62.9	46.3	30.8
**V2**	27.6	28.0	27.3	24.4	33.4	32.5	53.9	24.8	38.2	28.9	27.6	34.3	39.2	59.5	40.9	26.3
**VP**	28.0	16.8	17.8	17.4	22.4	22.0	48.3	16.3	28.2	20.3	27.4	27.7	30.2	54.1	31.2	19.9
**V3**	19.3	30.4	27.4	22.6	36.0	34.3	50.0	27.4	41.1	28.8	19.9	31.7	40.2	55.9	43.3	27.5
**V3A**	14.6	26.8	23.5	18.9	32.7	31.0	45.8	24.7	38.1	25.6	14.5	28.1	37.1	51.8	40.4	25.1
**MT**	16.0	16.3	13.1	8.2	21.6	19.5	37.9	15.8	26.6	14.2	15.5	18.1	25.5	43.9	28.4	17.1
**V4t**	18.6	14.8	11.6	9.9	21.1	19.2	40.3	17.1	27.0	15.7	18.0	20.0	27.0	46.4	29.0	18.9
**V4**	23.3	16.3	14.1	15.7	22.5	20.9	45.8	20.1	28.9	19.2	22.4	25.5	30.0	51.5	30.3	22.6
**VOT**	29.1	7.4	8.6	13.2	12.9	12.2	42.1	14.4	19.5	12.8	27.8	22.5	21.8	47.7	21.6	18.1
**MSTd**	12.4	21.5	18.7	9.1	25.7	23.2	36.4	20.2	30.1	16.5	11.0	16.0	27.3	42.4	31.6	20.6
**PIP**	13.6	27.6	24.8	18.0	32.4	30.5	42.9	23.5	36.7	24.6	14.5	27.2	35.3	49.0	39.0	22.4
**PO**	19.5	32.9	30.6	24.0	37.6	35.9	47.9	28.0	41.4	29.8	20.9	32.9	40.4	53.3	43.9	26.2
**DP**	10.1	31.9	28.2	22.7	37.7	35.7	46.2	29.8	42.7	29.6	11.5	30.8	40.9	52.0	44.7	29.4
**MIP**	18.2	34.0	31.4	24.2	38.8	37.0	45.4	29.5	42.5	30.6	20.0	33.0	41.0	50.8	44.7	28.2
**MDP**	21.1	38.7	35.9	28.3	43.1	41.0	46.7	33.7	46.7	34.2	23.2	36.6	44.5	52.0	48.9	31.4
**VIP**	7.4	28.8	25.7	16.9	33.1	30.7	36.8	25.9	36.5	23.8	9.4	24.3	33.6	42.6	38.1	24.9
**LIP**	0.0	31.2	28.0	18.7	35.5	33.1	39.0	28.9	39.5	26.2	7.1	25.5	36.2	45.1	40.9	28.2
**PITv**	31.2	0.0	8.3	13.9	9.3	8.3	40.5	14.1	15.4	11.2	29.7	21.6	18.4	45.8	17.2	18.1
**PITd**	28.0	8.3	0.0	12.1	12.7	10.4	39.9	15.8	19.1	10.9	26.5	20.2	20.2	45.4	19.7	19.2
**MSTl**	18.7	13.9	12.1	0.0	17.7	15.1	32.4	14.3	22.2	8.8	17.2	11.9	19.8	38.4	23.9	15.8
**CITv**	35.5	9.3	12.7	17.7	0.0	6.4	38.7	14.6	9.3	11.8	33.9	20.9	13.8	43.2	10.9	18.3
**CITd**	33.1	8.3	10.4	15.1	6.4	0.0	36.5	13.9	10.3	9.0	31.4	18.5	12.4	41.2	10.7	17.2
**FEF**	39.0	40.5	39.9	32.4	38.7	36.5	0.0	39.9	36.5	30.5	39.4	33.3	29.3	11.2	35.2	39.8
**TF**	28.9	14.1	15.8	14.3	14.6	13.9	39.9	0.0	16.4	12.7	27.9	21.7	19.0	44.7	19.6	9.7
**AITv**	39.5	15.4	19.1	22.2	9.3	10.3	36.5	16.4	0.0	14.3	38.3	21.7	10.7	39.9	7.4	18.5
**FST**	26.2	11.2	10.9	8.8	11.8	9.0	30.5	12.7	14.3	0.0	24.7	12.4	12.2	36.0	15.5	14.6
**7a**	7.1	29.7	26.5	17.2	33.9	31.4	39.4	27.9	38.3	24.7	0.0	23.6	35.3	45.4	40.0	27.6
**STPp**	25.5	21.6	20.2	11.9	20.9	18.5	33.3	21.7	21.7	12.4	23.6	0.0	16.0	38.4	22.2	23.3
**STPa**	36.2	18.4	20.2	19.8	13.8	12.4	29.3	19.0	10.7	12.2	35.3	16.0	0.0	33.1	10.2	20.7
**46**	45.1	45.8	45.4	38.4	43.2	41.2	11.2	44.7	39.9	36.0	45.4	38.4	33.1	0.0	38.3	44.6
**AITd**	40.9	17.2	19.7	23.9	10.9	10.7	35.2	19.6	7.4	15.5	40.0	22.2	10.2	38.3	0.0	21.8
**TH**	28.2	18.1	19.2	15.8	18.3	17.2	39.8	9.7	18.5	14.6	27.6	23.3	20.7	44.6	21.8	0.0

Table S5: Distances (in mm) between the areas of the model computed as the median of the distances between all vertex pairs of the two areas in their surface representation in F99 space, a standard macaque cortical surface included with Caret (Van Essen et al, 2001), where the vertex-to-vertex distance is the length of the shortest possible path without crossing the cortical surface (Bojak et al, 2011).

External input

Area	2/3E		2/3I		4E		4I		5E		5I		6E		6I	
V1	3,550	1,246	2,885	1,246	1,975	1,246	2,860	1,246	4,100	1,246	1,632	1,246	2,008	1,246	1,644	1,246
V2	3,608	1,848	3,853	1,848	3,413	1,848	4,819	1,848	5,669	1,848	3,124	1,848	4,596	1,848	3,511	1,848
VP	4,345	1,756	4,345	1,756	3,455	1,756	4,233	1,756	6,012	1,756	2,598	1,756	3,383	1,756	2,605	1,756
V3	4,227	1,810	4,270	1,810	3,833	1,810	4,664	1,810	6,341	1,810	2,576	1,810	3,618	1,810	2,558	1,810
V3A	6,086	2,703	6,347	2,703	7,114	2,703	8,001	2,703	7,881	2,703	3,714	2,703	4,786	2,703	3,587	2,703
MT	5,530	2,510	5,685	2,510	6,383	2,510	6,841	2,510	7,557	2,510	3,372	2,510	4,537	2,510	3,326	2,510
V4t	5,700	2,293	6,234	2,293	5,856	2,293	6,867	2,293	7,815	2,293	3,843	2,293	4,952	2,293	3,795	2,293
V4	4,749	2,337	5,074	2,337	5,481	2,337	5,861	2,337	7,051	2,337	3,272	2,337	4,769	2,337	3,453	2,337
VOT	5,065	2,409	5,346	2,409	7,426	2,409	9,952	2,409	5,375	2,409	2,786	2,409	3,713	2,409	2,462	2,409
MSTd	7,356	3,181	7,219	3,181	8,903	3,181	9,986	3,181	8,606	3,181	3,938	3,181	4,714	3,181	3,764	3,181
PIP	6,913	3,327	7,216	3,327	8,900	3,327	10,165	3,327	8,286	3,327	4,069	3,327	4,971	3,327	3,859	3,327
PO	7,482	3,226	7,432	3,226	8,083	3,226	8,943	3,226	9,001	3,226	4,167	3,226	4,879	3,226	4,033	3,226
DP	7,751	3,328	7,793	3,328	9,097	3,328	9,133	3,328	9,596	3,328	4,477	3,328	5,249	3,328	4,385	3,328
MIP	8,244	3,474	7,919	3,474	8,191	3,474	8,911	3,474	10,903	3,474	4,303	3,474	4,547	3,474	4,105	3,474
MDP	6,349	5,186	6,702	5,186	3,587	5,186	7,457	5,186	6,246	5,186	3,493	5,186	3,271	5,186	3,086	5,186
VIP	6,602	3,378	6,777	3,378	7,163	3,378	8,095	3,378	9,069	3,378	3,939	3,378	5,653	3,378	4,078	3,378
LIP	7,331	3,311	7,438	3,311	8,690	3,311	8,926	3,311	9,781	3,311	4,362	3,311	4,623	3,311	3,910	3,311
PITv	6,108	2,441	5,906	2,441	5,602	2,441	7,010	2,441	7,243	2,441	3,231	2,441	3,892	2,441	3,136	2,441
PITd	5,820	2,471	5,721	2,471	6,000	2,471	7,663	2,471	6,760	2,471	3,105	2,471	3,818	2,471	2,957	2,471
MSTl	7,491	3,094	7,482	3,094	8,566	3,094	9,595	3,094	8,935	3,094	4,122	3,094	5,013	3,094	3,917	3,094
CITv	8,696	3,844	8,567	3,844	12,863	3,844	13,354	3,844	9,926	3,844	4,627	3,844	5,434	3,844	4,387	3,844
CITd	7,641	3,708	8,066	3,708	17,442	3,708	20,485	3,708	8,023	3,708	4,204	3,708	5,357	3,708	3,714	3,708
FEF	7,499	3,597	7,936	3,597	9,253	3,597	9,708	3,597	8,286	3,597	4,003	3,597	4,634	3,597	3,802	3,597
TF	7,497	3,805	7,692	3,805	8,692	3,805	10,184	3,805	8,790	3,805	4,268	3,805	5,135	3,805	4,027	3,805
AITv	8,947	3,786	8,716	3,786	12,235	3,786	12,248	3,786	10,346	3,786	4,735	3,786	5,498	3,786	4,539	3,786
FST	9,905	4,614	10,189	4,614	14,721	4,614	15,183	4,614	11,516	4,614	5,671	4,614	6,641	4,614	5,428	4,614
7a	9,280	4,361	9,450	4,361	14,158	4,361	12,136	4,361	11,391	4,361	5,446	4,361	6,207	4,361	5,206	4,361
STPp	8,147	4,246	8,771	4,246	14,959	4,246	15,201	4,246	9,707	4,246	5,026	4,246	5,931	4,246	4,669	4,246
STPa	8,283	4,032	8,546	4,032	17,072	4,032	18,775	4,032	9,054	4,032	4,548	4,032	5,531	4,032	4,151	4,032
46	8,562	4,309	9,443	4,309	12,826	4,309	11,556	4,309	10,709	4,309	5,580	4,309	6,265	4,309	5,267	4,309
AITd	9,256	3,784	8,883	3,784	11,106	3,784	10,468	3,784	10,878	3,784	4,865	3,784	5,540	3,784	4,731	3,784
TH	9,229	5,491	9,829	5,491					9,468	5,491	4,774	5,491	6,566	5,491	5,629	5,491

Table S6: Numbers of intrinsic (light gray column) and extrinsic (dark gray column) synapses per neuron for all areas of the model.

Laminar thicknesses

Area	1	2/3	4	5	6	Total
V1	0.09	0.37	0.46	0.17	0.16	1.24
V2	0.12	0.60	0.24	0.25	0.25	1.46
VP	0.18	0.63	0.32	0.21	0.25	1.59
V3	0.23	0.70	0.31	0.16	0.19	1.59
V3A	0.20	0.71	0.24	0.23	0.28	1.66
MT	0.20	0.95	0.26	0.26	0.29	1.96
V4t	0.22	0.80	0.29	0.26	0.31	1.88
V4	0.18	1.00	0.24	0.24	0.24	1.89
VOT	0.23	0.81	0.28	0.27	0.32	1.90
MSTd	0.26	0.92	0.24	0.30	0.36	2.07
PIP	0.26	0.92	0.24	0.30	0.36	2.07
PO	0.26	0.92	0.24	0.30	0.36	2.07
DP	0.26	0.91	0.23	0.30	0.36	2.06
MIP	0.20	0.85	0.17	0.16	0.70	2.07
MDP	0.26	0.92	0.24	0.30	0.36	2.07
VIP	0.25	1.17	0.28	0.21	0.16	2.07
LIP	0.25	1.00	0.24	0.24	0.57	2.30
PITv	0.23	0.81	0.28	0.27	0.32	1.90
PITd	0.23	0.81	0.28	0.27	0.32	1.90
MSTl	0.26	0.92	0.24	0.30	0.36	2.07
CITv	0.29	1.02	0.19	0.33	0.40	2.23
CITd	0.29	1.02	0.19	0.33	0.40	2.23
FEF	0.22	0.92	0.35	0.37	0.35	2.21
TF	0.23	0.66	0.21	0.24	0.28	1.62
AITv	0.34	1.20	0.23	0.39	0.47	2.63
FST	0.51	0.90	0.18	0.30	0.36	2.25
7a	0.35	1.24	0.21	0.41	0.48	2.68
STPp	0.29	1.03	0.18	0.34	0.40	2.25
STPa	0.29	1.03	0.18	0.34	0.40	2.25
46	0.22	0.82	0.18	0.28	0.36	1.86
AITd	0.34	1.20	0.23	0.39	0.47	2.63
TH	0.28	0.65	0.12	0.57	0.26	1.87

Table S7: Laminar thicknesses in mm for all 32 areas of the model. Values are rounded to two decimal places. These values are used to determine population sizes for the modeled layers 2/3, 4, 5 and 6 and to distribute synapses across layers 1 to 6 of target areas for cortico-cortical connections (cf. Results and Table S11).

Area surfaces

Area	Surface area (mm^2)	Area	Surface area (mm^2)	Area	Surface area (mm^2)
V1	1484.63	V3	120.57	PO	75.37
V2	1193.40	CITv	114.67	VOT	70.11
V4	561.41	DP	113.83	FST	61.33
STPp	245.48	PIP	106.15	CITd	57.54
TF	197.40	PITv	100.34	LIP	56.04
46	185.16	V3A	96.96	MT	55.90
FEF	161.54	AITv	93.12	MIP	45.09
7a	157.34	AITd	91.59	TH	44.60
PITd	145.38	VIP	85.06	MSTl	29.19
VP	130.58	STPa	78.72	V4t	28.23
MSTd	120.57	MDP	77.49		

Table S8: Surface areas computed with Caret (Van Essen et al, 2001) on the basis of each area's representation on the F99 cortical surface (Van Essen, 2002). Areas are ordered from large to small.

Population sizes

Area	2/3E	2/3I	4E	4I	5E	5I	6E	6I	Total
V1	47,386	13,366	70,387	17,597	20,740	4,554	19,839	4,063	197,935
V2	50,521	14,250	36,685	9,171	19,079	4,189	19,248	3,941	157,087
VP	52,973	14,942	49,292	12,323	15,929	3,497	19,130	3,917	172,007
V3	58,475	16,494	47,428	11,857	12,056	2,647	14,529	2,975	166,465
V3A	40,887	11,532	23,789	5,947	12,671	2,782	15,218	3,116	115,946
MT	60,606	17,095	28,202	7,050	14,176	3,113	15,837	3,243	149,324
V4t	48,175	13,588	34,735	8,684	14,857	3,262	17,843	3,654	144,801
V4	64,447	18,178	33,855	8,464	13,990	3,072	14,161	2,900	159,070
VOT	45,313	12,781	37,611	9,403	15,828	3,475	19,008	3,892	147,315
MSTd	44,343	12,507	22,524	5,631	14,742	3,237	17,704	3,625	124,318
PIP	44,343	12,507	22,524	5,631	14,742	3,237	17,704	3,625	124,318
PO	44,343	12,507	22,524	5,631	14,742	3,237	17,704	3,625	124,318
DP	43,934	12,392	18,896	4,724	14,179	3,113	17,028	3,487	117,755
MIP	41,274	11,642	15,875	3,969	7,681	1,686	34,601	7,086	123,816
MDP	44,343	12,507	22,524	5,631	14,742	3,237	17,704	3,625	124,318
VIP	56,683	15,988	26,275	6,569	10,099	2,217	7,864	1,610	127,310
LIP	51,983	14,662	20,095	5,024	11,630	2,554	28,115	5,757	139,824
PITv	45,313	12,781	37,611	9,403	15,828	3,475	19,008	3,892	147,315
PITd	45,313	12,781	37,611	9,403	15,828	3,475	19,008	3,892	147,315
MSTl	44,343	12,507	22,524	5,631	14,742	3,237	17,704	3,625	124,318
CITv	41,696	11,761	15,303	3,826	14,385	3,158	17,275	3,537	110,944
CITd	41,696	11,761	15,303	3,826	14,385	3,158	17,275	3,537	110,944
FEF	44,053	12,425	23,143	5,786	16,943	3,720	16,128	3,302	125,504
TF	30,774	8,680	17,143	4,286	11,082	2,433	13,310	2,725	90,436
AITv	49,224	13,884	18,066	4,516	16,982	3,729	20,395	4,176	130,977
FST	36,337	10,249	12,503	3,126	12,624	2,772	15,160	3,104	95,879
7a	49,481	13,957	13,279	3,320	15,817	3,473	18,996	3,890	122,216
STPp	41,677	11,755	13,092	3,273	14,218	3,122	17,075	3,496	107,712
STPa	41,677	11,755	13,092	3,273	14,218	3,122	17,075	3,496	107,712
46	32,581	9,190	10,645	2,661	11,850	2,602	15,841	3,244	88,617
AITd	49,224	13,884	18,066	4,516	16,982	3,729	20,395	4,176	130,977
TH	24,712	6,970			23,353	5,128	10,861	2,224	73,251

Table S9: Estimated population sizes across layers and areas underneath $1\,\mathrm{mm}^2$ of cortical surface in each area.

Derivation of local average connection probability

The average connection probability is obtained by integrating over all possible positions of the two neurons,

$$\bar{C}(R) = \frac{C_0}{\pi^2 R^4} \int_0^R \int_0^{2\pi} \int_0^R \int_0^{2\pi} \exp\left[\frac{-\left(r_1^2 + r_2^2 - 2r_1 r_2 \cos(\theta_1 - \theta_2)\right)}{2\sigma^2}\right] r_1 r_2 \mathrm{d}\theta_1 \mathrm{d}r_1 \mathrm{d}\theta_2 \mathrm{d}r_2 \,, \tag{1}$$

with C_0 the connection probability at zero distance and polar coordinates $r_1, r_2, \theta_1, \theta_2$. This can be reduced to a simpler form (Sheng, 1985),

$$\bar{C}(R) = \frac{2C_0}{\pi R^2} \int_0^{2R} e^{-r^2/2\sigma^2} \left[4 \arctan\left(\frac{2R - r}{2R + r}\right)^{1/2} - \sin\left(4 \arctan\left[\frac{2R - r}{2R + r}\right]^{1/2}\right)\right] r \mathrm{d}r \,. \tag{2}$$

Local connectivity

The indegrees of the microcircuit model (Potjans and Diesmann, 2014) $K'_{ij}(R)$ are adapted to the area-specific laminar compositions of the multi-area model with an area-specific factor $c_A(R)$,

$$K_{ij}(R) = c_A(R) K'_{ij}(R) \;\; \forall i, j \,,$$

where i, j denote single populations in the $1\,\mathrm{mm}^2$ patch of the cortical area. The total number of synapses local to the patch (type I) is the sum over the projections between all populations of the area:

$$N^{\mathrm{syn,I}} = \sum_{i,j} N_i K_{ij} = c_A \sum_{i,j} N_i K'_{ij} \,.$$

We thus obtain $c_A(R)$ by determining $N^{\mathrm{syn,I}}$. To this end, we use retrograde tracing data from Markov et al (2011) consisting of fractions of labeled neurons (FLN) per area as a result of injections into one area at a time. The fraction intrinsic to the injected area, FLN_{i}, is approximately equal for all 9 areas where this fraction was determined, with a mean of 0.79. For areas modeled with reduced size, this fraction is smaller because, in that case, synapses of both type I and II contribute to the value of 0.79 (Fig. 4E). We approximate the increasing

contribution of type I synapses with the modeled area size as the increase in indegrees averaged over population pairs,

$$\frac{N^{\mathrm{syn,I}}(R)/N^{\mathrm{syn,tot}}(R)}{N^{\mathrm{syn,I}}(R_{\mathrm{full}})/N^{\mathrm{syn,tot}}(R_{\mathrm{full}})} = \left\langle \frac{K_{ij}(R)}{K_{ij}(R_{\mathrm{full}})} \right\rangle_{ij} = \left\langle \frac{K'_{ij}(R)}{K'_{ij}(R_{\mathrm{full}})} \right\rangle_{ij},$$

where in the last step we use eq. (7). Using $N^{\mathrm{syn,I}}(R_{\mathrm{full}})/N^{\mathrm{syn,tot}}(R_{\mathrm{full}}) = FLN_{\mathrm{i}}$, we obtain

$$N^{\mathrm{syn,I}}(R) = N^{\mathrm{syn,tot}}(R)\, FLN_{\mathrm{i}} \left\langle \frac{K'_{ij}(R)}{K'_{ij}(R_{\mathrm{full}})} \right\rangle_{ij},$$

where $N^{\mathrm{syn,tot}}(R) = \rho_{\mathrm{syn}} \pi R^2 D$ with D the total thickness of the given area. The conversion factor can thus be obtained with

$$c_A(R) = \frac{N^{\mathrm{syn,tot}}(R)}{\sum_{i,j} N_i K'_{ij}}\, FLN_{\mathrm{i}} \left\langle \frac{K'_{ij}(R)}{K'_{ij}(R_{\mathrm{full}})} \right\rangle_{ij}.$$

We substitute this into eq. (7) for the modeled areas where $R = R_0$ and obtain the population-specific indegrees for type I synapses:

$$K_{ij,\mathrm{I}} := K_{ij}\,(R = R_0)$$

Processing of CoCoMac data

We use a new release of CoCoMac, in which mappings from brain regions in other nomenclatures were scrutinized to ensure a consistent transfer of connections into the FV91 name space. The CoCoMac database provides information on laminar patterns on the source side from retrograde tracing studies as well as on the target side from anterograde tracing studies. The data was extracted by using the following link, which specifies all search options: `http://cocomac.g-node.org/cocomac2/services/connectivity_matrix.php?dbdate=20141022&AP=AxonalProjections_FV91&constraint=&origins=&terminals=&square=1&merge=max&laminar=both&format=json&cite=1`

Furthermore, we obtained the numbers of confirmative studies for each area-level connection with the following link: `http://cocomac.g-node.org/cocomac2/services/connectivity_matrix.php?dbdate=20141022&AP=AxonalProjections_FV91&constraint=&origins=&terminals=&square=1&merge=count&laminar=off&format=json&cite=1`

To process these data, we applied the following steps:

- A connection is assumed to exist if there is at least one confirmative study reporting it.
- A connection from layer 2/3 is modeled if CoCoMac indicates a connection from either or both of layers 2 and 3.
- In the database, some layers carry an 'X' indicating a connection of unknown strength. We interpret these as '2' (corresponding to medium connection strength).
- We take connection strengths in CoCoMac to represent numbers of synapses in orders of magnitude, i.e., the relative number of synapses N^{ν}_{syn} in layer ν of area A with connection strength $\alpha(\nu)$ is computed as $N^{\nu}_{\mathrm{syn}} = 10^{\alpha(v)} / \sum_{v' \in A} 10^{\alpha(\nu')}$.

Mapping of synapse to cell-body locations

Detailed calculation in section Materials and Methods. The numbers are listed in Table S11. We map the 17 different cell types of Binzegger et al (2004) to the 8 cortical populations considered in our network using Table S10.

Population in the network	Binzegger et al (2004)
2/3E	p2/3
2/3I	b2/3, nb2/3
4E	ss4 (L4), ss4 (L2/3), p4
4I	b4, nb4
5E	p5 (L2/3), p5 (L5/6)
5I	b5, nb5
6E	p6 (L4), p6 (L5/6)
6I	b6, nb6

Table S10: Mapping of Binzegger et al (2004) cell types to the 8 populations of the cortical areas in the network.

Target population	Synapse layer: 1	2/3	4	5	6
2/3E	0.57				
2/3I		0.16			
4E	0.18	0.84	0.73		
4I			0.16		
5E	0.25		0.02	0.76	
5I				0.1	
6E	0.003		0.09	0.14	0.85
6I					0.15

Table S11: Conditional probabilities $\mathcal{P}(i|s_{\mathrm{cc}} \in v)$ for the target neuron to belong to population i if a cortico-cortical synapse s_{cc} is located in layer v, computed with eq. (11) applied to the data set of Binzegger et al (2004). Empty cells signal zero probabilities.

Derivation of the cortico-cortical connectivity

The number of cortico-cortical synapses from excitatory population j in area B to population i in area A is calculated as

$$N^{\mathrm{syn,III}}(A,i,B,j) = \underbrace{Z_i \sum_{v \in P_{\mathrm{t}}} Y_v \, \mathcal{P}(i|s_{\mathrm{cc}} \in v)}_{\text{target side}} \underbrace{X_j}_{\text{source side}} N^{\mathrm{syn,III}}(A,B)\,, \tag{3}$$

$$\text{with } X_j = \begin{cases} SLN_{AB} & \text{if } j \in S \bigcap P_{\mathrm{s}} \\ (1 - SLN_{AB}) \frac{10^{\alpha(v_j)}}{\sum_{j' \in I,\, \alpha(v_{j'})>0} 10^{\alpha(v_{j'})}} & \text{if } j \in I \text{ and } \alpha(v_j) > 0 \\ (1 - SLN_{AB}) \frac{N_{jB}}{\sum_{j' \in I} N_{j'B}} & \text{if } j \in I \bigcap P_{\mathrm{s}} \text{ but no CoCoMac data available} \\ 0 & \text{if } j \notin P_{\mathrm{s}} \end{cases},$$

$$\text{and } Y_v = \begin{cases} \frac{10^{\alpha(v_i)}}{\sum_{\alpha(v')>0} 10^{\alpha(v')}} & \text{if } \alpha(v) > 0 \\ \frac{D_{Av_i}}{\sum_{v'} D_{Av'}} & \text{if no CoCoMac data available} \end{cases}.$$

Here, $S = 2/3\mathrm{E}$ and $I = \{5\mathrm{E},6\mathrm{E}\}$ respectively denote the supragranular and infragranular excitatory populations, v_i is the layer containing population i, and $\alpha(v)$ is the layer-specific labeling density estimate from CoCoMac. Z_i is a factor which takes into account that cortico-cortical feedback connections preferentially target excitatory rather than inhibitory neurons (Johnson and Burkhalter, 1996; Anderson et al, 2011). For each feedback connection in the model, we redistribute the synapses across the excitatory and inhibitory target populations and determine Z_i such that 93 % of synapses in each cortico-cortical projection are established on excitatory neurons.

Validation of network characteristics

To validate our findings, we tested the main characteristics of the network model against alterations of three main heuristics used in its construction:

Exponential decay of connection densities

To test the robustness of the area-level connectivity, we excluded a random subset of the FLN data (Markov et al, 2014a) and repeated the fit of the exponential decay of connection densities (Fig. 4C) for these reduced datasets (Figure S4A). We then used the altered fitted parameters to estimate both unknown and excluded data and proceeded with the network construction. To test if this had a significant effect on the network, we repeated the clustering analysis of Fig. 7 for 50 different trials (Figure S4B). The community structure is generally stable if 5% or 10% of the data are ignored. Ventral and dorsal stream areas are assigned to separate large clusters. Frontal areas are consistently clustered together. The two lower visual areas V1 and V2 are clustered together except in two trials, where the data on their connectivity is ignored so that it is estimated using the EDR rule, resulting in a lower connection density than suggested by the data. Areas STPp, STPa, PITd, and MSTd change clusters frequently. However, overall, the community structure proves to be robust against leaving out small portions of the underlying experimental data. If half of the experimental data is excluded, the resulting fits for the exponential decay of connection densities show clear deviations. Still, ventral and dorsal stream areas as well as frontal areas are well separated into clusters, but the cluster assignment of the remaining areas varies across trials. As an additional validation, we used the full FLN data to fit the exponential decay of connection densities and then introduced a random offset uniformly distributed within $\pm\varDelta$ for the logarithm of each estimated FLN to reflect the spread of experimental data around the fitted exponential decay of connection densities. We chose $\varDelta = 1.55$ to be the median of the absolute deviation of the logarithm of experimental data and exponential fit. This leads to a degree of variation between trials that is comparable to the case of pruning 10% of the data. Areas PITd and MSTd frequently and FST, STPa, STPp, VP, and VOT to a lesser extent change clusters.

In conclusion, the community structure of the network does not rely on single data points, but the data leave open the possibility of different cluster assignments especially of areas PITd and MSTd.

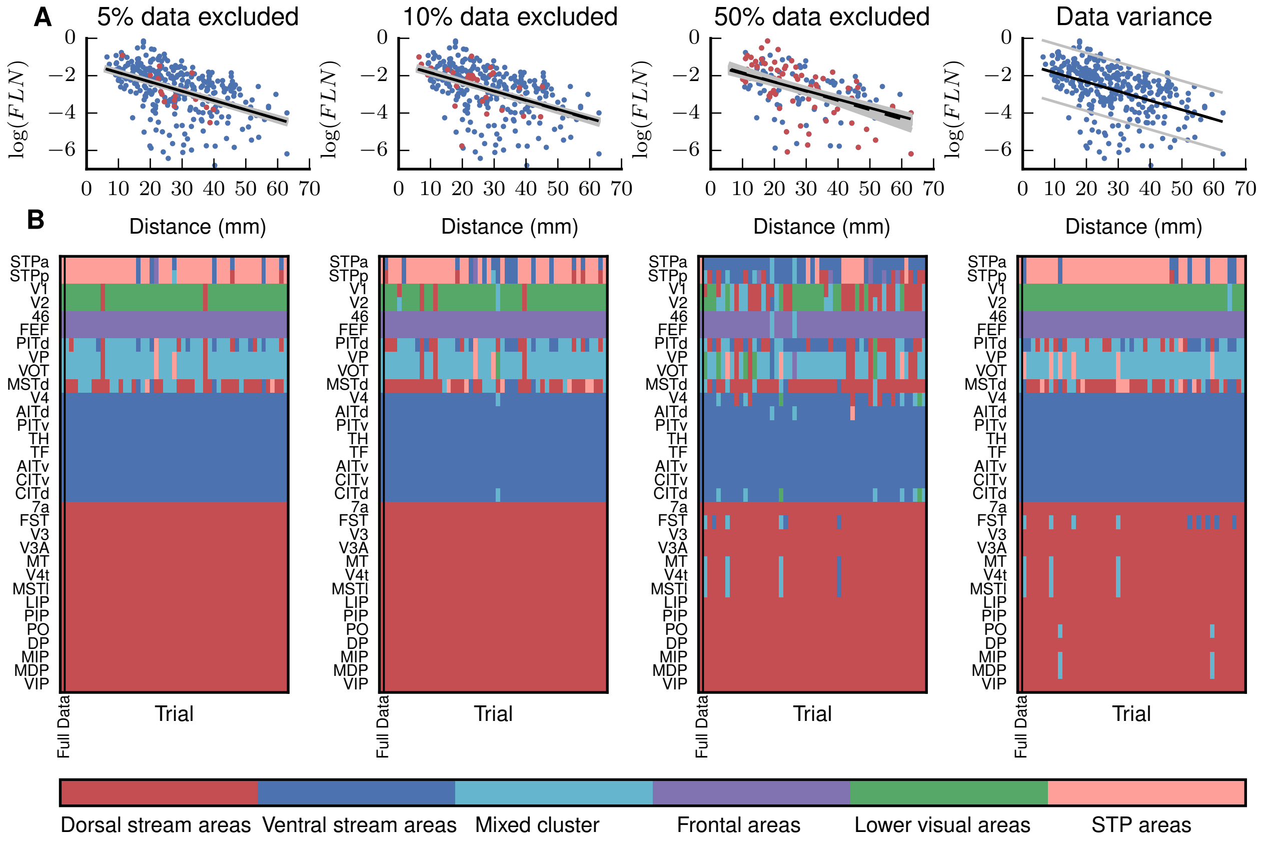

Figure S4: **Robustness of modular network structure** against using only fractions of the experimental data and increasing the variance of the estimated FLN. **a** Experimentally measured FLN from Markov et al (2014a) versus inter-areal distance. Same display as in 4C. Black line: Linear regression using a randomly drawn subset of data points (blue dots) and ignoring the remaining data points (red dots). Overlapping gray lines show linear regression lines for different trials (first three columns). The gray lines in the last column indicate the upper and lower boundary ($\Delta(\log FLN \in [-1.55, 1.55])$ of the random offset. The dashed line (overlapping with black line) shows the regression using the full dataset (as shown in Fig. 4C). **b** Visualization of community structure in the resulting network connectivity using the fitted parameters for the exponential decay of connection densities on randomly drawn subsets of the data. Clusters are determined by applying the map equation method (Rosvall et al, 2009) on the outdegrees, as in Fig. 7. The colors and cluster names are chosen as in Fig. 7. For comparison, the first column of each matrix shows the clustering of the network based on the full dataset as shown in Fig. 7

Threshold for hierarchical categorization based on SLN

In Fig. 5B, we took connections with $SLN < 0.35$ to correspond to feedback projections, $SLN > 0.65$ to feedforward projections and $SLN \in [0.35, 0.65]$ to lateral projections. To test whether the choice of these thresholds has a crucial influence, we tested for two different thresholds of [0.3, 0.7] and [0.4, 0.6] if the resulting laminar patterns in the connectivity and the shortest paths between areas were significantly altered. Figure S5 shows that the chosen threshold has little influence on the laminar connectivity patterns in the network. Both the target patterns and the resulting population-specific connectivity patterns show only marginal differences. The laminar distribution of shortest paths reveals small differences depending on the chosen threshold: For a broader range of SLN corresponding to lateral connections (middle column), feedforward paths solely originate in layer 2/3. For a narrower range of SLN corresponding to lateral connections (right column), more feedback connections are formed in layer 2/3. The qualitative picture remains unchanged: There are clear differences between feedforward and feedback connections, and lateral connections are more similar to feedforward connections than to feedback connections in their start-end patterns.

Figure S5: **Laminar connectivity patterns and shortest paths are robust against altering SLN threshold. Top panels**: Same display as in Fig. 5B. Laminar target patterns of synapse locations in relation to the fraction of supragranular labeled neurons (SLN) of the source pattern. Target patterns are taken from the CoCoMac database (Felleman and Van Essen, 1991; Barnes and Pandya, 1992; Suzuki and Amaral, 1994; Morel and Bullier, 1990; Perkel et al, 1986; Seltzer and Pandya, 1994) and SLN data from Markov et al (2014b) mapped to the FV91 scheme. **Middle panels:** Same display as in Fig. 5D. Laminar patterns of cortico-cortical connections in the feedback, lateral, and feedforward direction, measured as the indegree of the population pairs divided by the sum of indegrees over all pairs, and then averaged across area pairs with the respective connection type ($K_{ij} = \langle K_{iA,jB} / \sum_{i',j'} K_{i'A,j'B} \rangle_{A,B}$). The categorization into feedback, lateral, and feedforward types follows the SLN value as in the upper row. **Bottom panels:** Same display as in Fig. 8A. Population-specific patterns of shortest paths between directly connected pairs of areas categorized according to their hierarchical relation as defined by the SLN. Arrow thickness indicates the occurrence of the particular pattern.

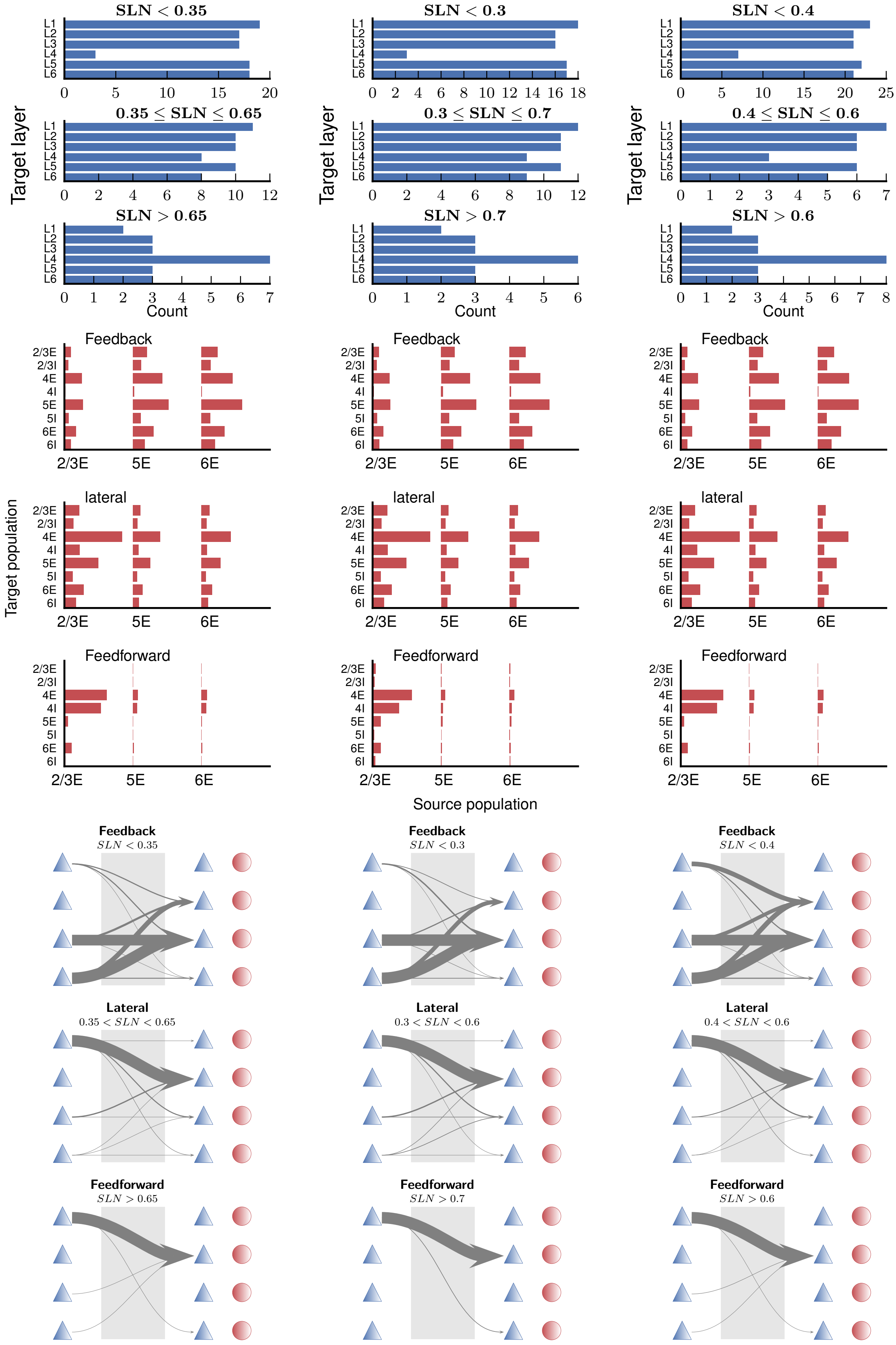

Figure S5: **Laminar connectivity patterns and shortest paths are robust against altering SLN threshold.**

Sigmoidal relation of SLN with log cell density ratios

To test the robustness of our path analysis (Fig. 8) against the sigmoidal relation of SLN versus logarithmic cell density ratios, we used a similar procedure as for the exponential decay of connection densities: We ignored random subsets of the data (5%, 10% and 50%) from Markov et al (2014b) for the sigmoidal fit and then estimated missing data as well as the ignored data based on this modified fit. The fit of the sigmoid is only slightly influenced by excluding subsets of the data (Figure S6A). The resulting patterns of shortest paths also show little variation across trials. For 50% of data pruning, there is visible variation in the results, but the qualitative picture still holds. This shows that the heuristic of the SLN sigmoid delivers a robust basis for laminar connection patterns in the network model.

Figure S6: **Laminar connectivity patterns and shortest paths are robust against altering SLN threshold.** We randomly choose 5% (top part), 10% (middle part), and 50% (bottom part) of the SLN data of Markov et al (2014b) and repeat this for 50 trials. **Left part** Same display as in Fig. 5A. Fraction of source neurons in supragranular layers (SLN) vs. logarithmized ratio of the overall neuron densities of the two areas. SLN from Markov et al (2014b), neuron densities from Hilgetag et al (2016b). Black curve, fit using a beta-binomial model eq. (1) on a randomly drawn subset of data points (blue dots) and ignoring the remaining data points (red dots). Overlapping gray lines show linear regression lines for different trials and the dashed line (overlapping with black line) shows the regression using the full dataset (as shown in Fig. 5C). **Right part:** Same display as Fig. 8. **Top panels** Population-specific patterns of shortest paths between directly connected pairs of areas categorized according to their hierarchical relation as defined by the SLN. Arrow thickness indicates the occurrence of the particular pattern. **Middle panels** Population-specific patterns of shortest paths between all pairs of areas categorized according to the difference between their architectural types. Arrow thickness indicates the relative occurrence of the particular pattern. In top and middle panels, gray arrows show the mean value across trials and the underlying red arrows show the mean plus one standard deviation (partly not visible because of the small variation across trials). **Lower panels** Occurrence of population patterns in areas that appear in the intermediate stage in the shortest path between two areas. Error bars indicate standard deviation across trials (partly not visible because of small values)

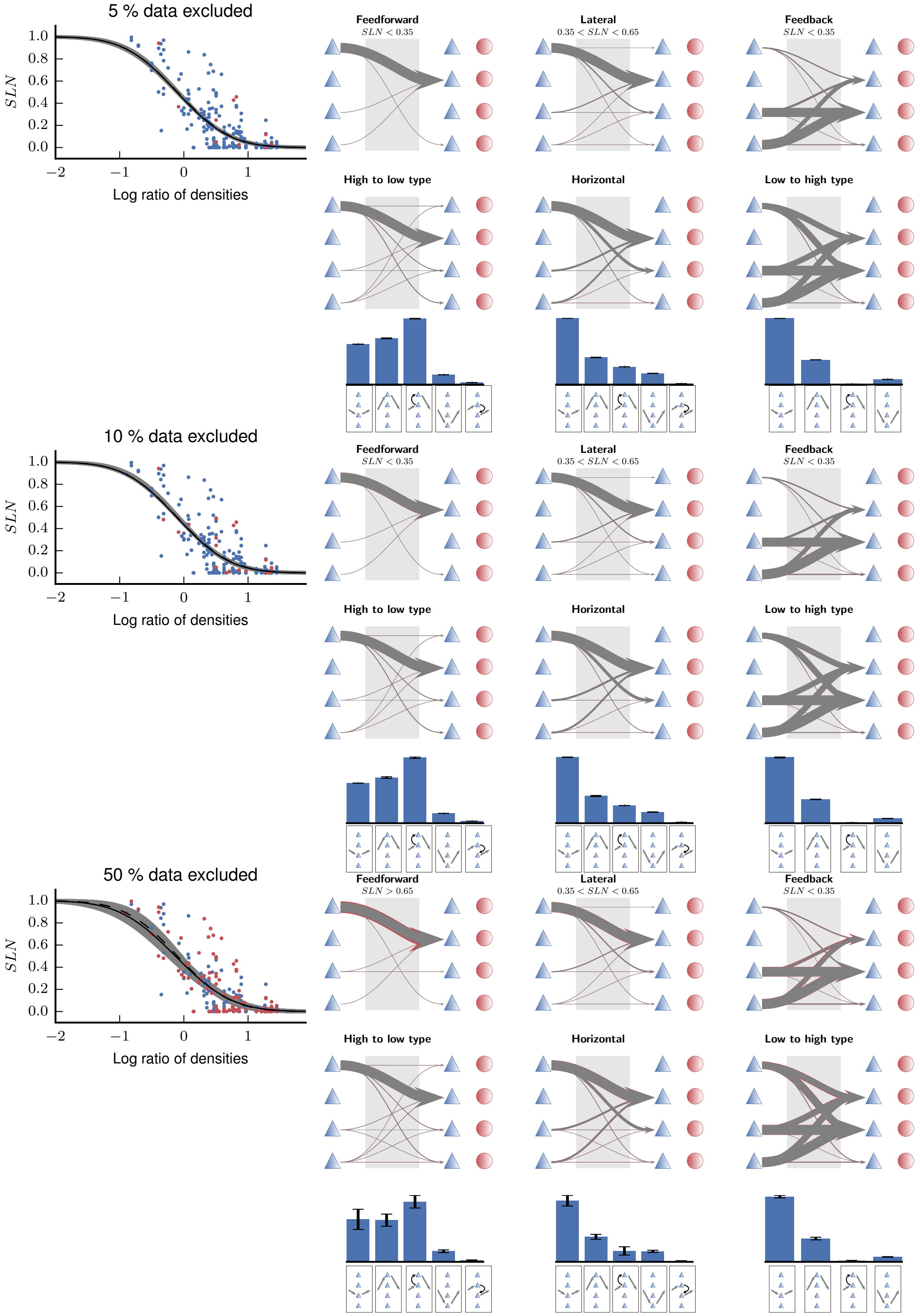

Figure S6: **Laminar patterns of shortest paths are robust against using only a subset of SLN data.**

Path analysis using only connections with experimental SLN

To test whether the predicted laminar pattern of shortest paths depend crucially on the SLN predicted with the sigmoid, we repeated the path analysis shown in Fig. 8 but took only connections into account for which SLN data were provided by the dataset of Markov et al (2014b). This amounts to approx. 23% of the connections. Figure S7 shows that the laminar patterns of shortest paths largely match those determined from data plus estimates. The most obvious differences occur in low-to-high-type connections with a smaller proportion of paths starting in supragranular layers, and in horizontal connections with a larger proportion of paths starting in infragranular layers. Furthermore, layer 6 becomes more prominent compared to layer 5 in the origin of horizontal, feedback, and low-to-high-type paths. This does not reflect a shortcoming of the sigmoidal fit, but occurs because for this subset of connections there are relatively more source patterns stored in CoCoMac where L6 sends more connections than L5 than if all connections are taken into account. The overall characteristics, however, are still preserved, showing that the laminar patterns predicted by the model are not artificially introduced by the estimation of SLN through the sigmoidal relationship (Fig. 5A).

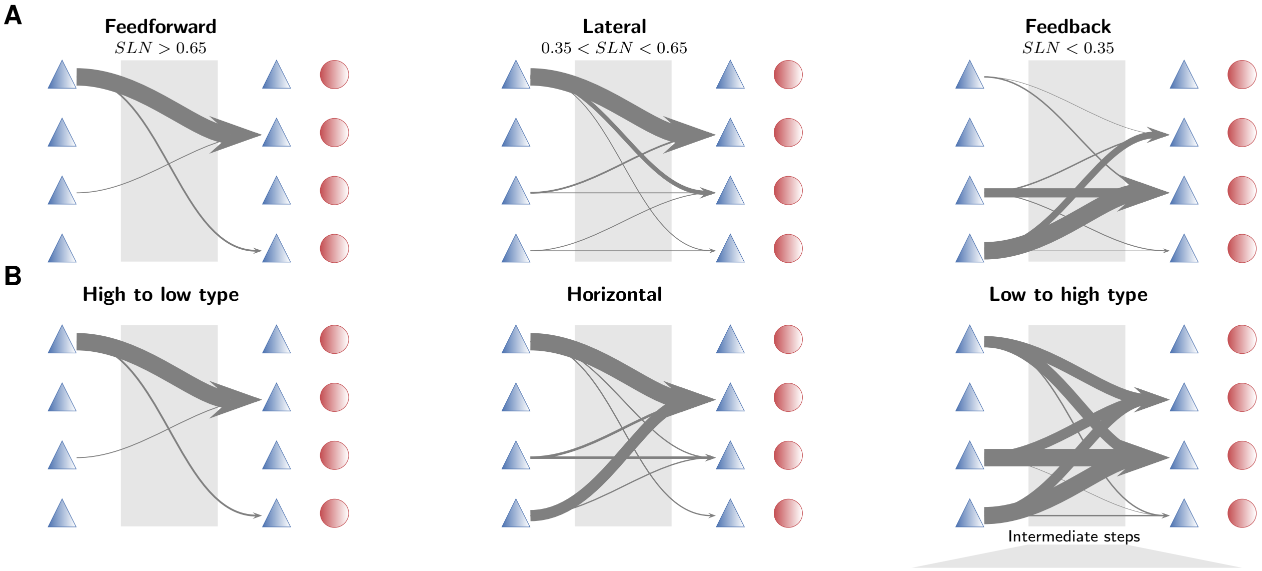

Figure S7: **Laminar patterns of shortest paths are not introduced by estimated SLN.** Same display as in Fig. 8 but taking only connections with available experimental data on fractions of supragranular labeled neurons (SLN)**. a** Population-specific patterns of shortest paths between directly connected pairs of areas categorized according to their hierarchical relation as defined by the SLN. Arrow thickness indicates the relative occurrence of the particular pattern. **b** Population-specific patterns of shortest paths between all pairs of areas categorized according to the difference between their architectural types. Arrow thickness indicates the occurrence of the particular pattern.

Supplemental References

Anderson JC, Kennedy H, Martin KAC (2011) Pathways of attention: Synaptic relationships of frontal eye field to V4, lateral intraparietal cortex, and area 46 in macaque monkey. J Neurosci 31(30):10,872–10,881, DOI 10.1523/JNEUROSCI.0622-11.2011, URL http://www.jneurosci.org/content/31/30/10872.abstract, http://www.jneurosci.org/content/31/30/10872.full.pdf+html

Angelucci A, Levitt JB, Lund JS (2002) Anatomical origins of the classical receptive field and modulatory surround field of single neurons in macaque visual cortical area V1. Prog Brain Res 136:373–388

Barnes CL, Pandya DN (1992) Efferent cortical connections of multimodal cortex of the superior temporal sulcus in the rhesus monkey. J Compar Neurol 318(2):222–244

Binzegger T, Douglas RJ, Martin KAC (2004) A quantitative map of the circuit of cat primary visual cortex. J Neurosci 39(24):8441–8453

Bojak I, Oostendorp TF, Reid AT, Kötter R (2011) Towards a model-based integration of co-registered electroencephalography/functional magnetic resonance imaging data with realistic neural population meshes. Phil Trans R Soc A 369(1952):3785–3801

Boussaoud D, Ungerleider L, Desimone R (1990) Pathways for motion analysis: Cortical connections of the medial superior temporal and fundus of the superior temporal visual areas in the macaque. J Compar Neurol 296(3):462–495

Eggan S, Lewis D (2007) Immunocytochemical distribution of the cannabinoid CB1 receptor in the primate neocortex: A regional and laminar analysis. Cereb Cortex 17(1):175–191

Felleman D, Burkhalter A, Van Essen D (1997) Cortical connections of areas V3 and VP of macaque monkey extrastriate visual cortex. J Compar Neurol 379(1):21–47

Felleman DJ, Van Essen DC (1991) Distributed hierarchical processing in the primate cerebral cortex. Cereb Cortex 1:1–47

Hilgetag CC, Medalla M, Beul S, Barbas H (2016a) The primate connectome in context: principles of connections of the cortical visual system. NeuroImage p (in press)

Hilgetag CC, Medalla M, Beul SF, Barbas H (2016b) The primate connectome in context: Principles of connections of the cortical visual system. NeuroImage 134:685–702

Johnson RR, Burkhalter A (1996) Microcircuitry of forward and feedback connections within rat visual cortex. J Compar Neurol 368:383–398

Lavenex P, Suzuki W, Amaral D (2002) Perirhinal and parahippocampal cortices of the macaque monkey: Projections to the neocortex. J Compar Neurol 447(4):394–420

Markov NT, Misery P, Falchier A, Lamy C, Vezoli J, Quilodran R, Gariel MA, Giroud P, Ercsey-Ravasz M, Pilaz LJ, Huissoud C, Barone P, Dehay C, Toroczkai Z, Van Essen DC, Kennedy H, Knoblauch K (2011) Weight consistency specifies regularities of macaque cortical networks. Cereb Cortex 21(6):1254–1272

Markov NT, Ercsey-Ravasz MM, Ribeiro Gomes AR, Lamy C, Magrou L, Vezoli J, Misery P, Falchier A, Quilodran R, Gariel MA, Sallet J, Gamanut R, Huissoud C, Clavagnier S, Giroud P, Sappey-Marinier D, Barone P, Dehay C, Toroczkai Z, Knoblauch K, Van Essen DC, Kennedy H (2014a) A weighted and directed interareal connectivity matrix for macaque cerebral cortex. Cereb Cortex 24(1):17–36, DOI 10.1093/cercor/bhs270, URL http://cercor.oxfordjournals.org/content/24/1/17.abstract, http://cercor.oxfordjournals.org/content/24/1/17.full.pdf+html

Markov NT, Vezoli J, Chameau P, Falchier A, Quilodran R, Huissoud C, Lamy C, Misery P, Giroud P, Ullman S, Barone P, Dehay C, Knoblauch K, Kennedy H (2014b) Anatomy of hierarchy: Feedforward and feedback pathways in macaque visual cortex. J Compar Neurol 522(1):225–259, DOI 10.1002/cne.23458, URL http://dx.doi.org/10.1002/cne.23458

Morel A, Bullier J (1990) Anatomical segregation of two cortical visual pathways in the macaque monkey. Visual neuroscience 4(06):555–578

O'Kusky J, Colonnier M (1982) A laminar analysis of the number of neurons, glia, and synapses in the visual cortex (area 17) of adult macaque monkeys. J Compar Neurol 210(3):278–290

Perkel DJ, Bullier J, Kennedy H (1986) Topography of the afferent connectivity of area 17 in the macaque monkey: A double-labelling study. J Compar Neurol 253(3):374–402

Petrides M, Pandya D (1999) Dorsolateral prefrontal cortex: comparative cytoarchitectonic analysis in the human and the macaque brain and corticocortical connection patterns. Eur J Neurosci 11(3):1011–1036

Potjans TC, Diesmann M (2014) The cell-type specific cortical microcircuit: Relating structure and activity in a full-scale spiking network model. Cereb Cortex 24(3):785–806, DOI doi:10.1093/cercor/bhs358

Preuss TM, Goldman-Rakic PS (1991) Myelo-and cytoarchitecture of the granular frontal cortex and surrounding regions in the strepsirhine primate galago and the anthropoid primate macaca. J Compar Neurol 310(4):429–474

Rakic P, Suñer I, Williams R (1991) A novel cytoarchitectonic area induced experimentally within the primate visual cortex. Proc Nat Acad Sci USA 88(6):2083–2087

Rockland K (1992) Configuration, in serial reconstruction, of individual axons projecting from area V2 to V4 in the macaque monkey. Cereb Cortex 2(5):353–374

Rosvall M, Axelsson D, Bergstrom CT (2009) The map equation. Eur Phys J Spec Top 178(1):13–23

Rozzi S, Calzavara R, Belmalih A, Borra E, Gregoriou G, Matelli M, Luppino G (2006) Cortical connections of the inferior parietal cortical convexity of the macaque monkey. Cereb Cortex 16(10):1389–1417

Seltzer B, Pandya DN (1994) Parietal, temporal, and occipita projections to cortex of the superior temporal sulcus in the rhesus monkey: A retrograde tracer study. J Compar Neurol 343(3):445–463

Sheng T (1985) The distance between two random points in plane regions. Adv Appl Prob 17(4):748–773

Suzuki WA, Amaral DG (2003) Perirhinal and parahippocampal cortices of the macaque monkey: cytoarchitectonic and chemoarchitectonic organization. J Compar Neurol 463(1):67–91

Suzuki WL, Amaral DG (1994) Perirhinal and parahippocampal cortices of the macaque monkey: cortical afferents. J Compar Neurol 350(4):497–533

Van Essen DC (2002) Windows on the brain: the emerging role of atlases and databases in neuroscience. Curr Opin Neurobiol 12(5):574–579

Van Essen DC (2012) Cortical cartography and caret software. NeuroImage 62(2):757–764

Van Essen DC, Drury HA, Dickson J, Harwell J, Hanlon D, Anderson CH (2001) An integrated software suite for surface-based analyses of cerebral cortex. J Am Med Inform Assoc 8(5):443–459
